# The Impact of C_2_ Insertion into a Carbazole Donor on the Physicochemical Properties of Dibenzo[*a,j*]phenazine‐Cored Donor–Acceptor–Donor Triads

**DOI:** 10.1002/chem.202101654

**Published:** 2021-08-13

**Authors:** Paola Zimmermann Crocomo, Takahito Kaihara, Soki Kawaguchi., Patrycja Stachelek, Satoshi Minakata, Piotr de Silva, Przemyslaw Data, Youhei Takeda

**Affiliations:** ^1^ Faculty of Chemistry Silesian University of Technology M. Strzody 9 44-100 Gliwice Poland; ^2^ Department of Applied Chemistry Graduate School of Engineering Osaka University Yamadaoka 2–1 Suita Osaka 565-0871 Japan; ^3^ Nada Junior and Senior High School Uozakikitamachi 8–5-1 Kobe Higashinada-ku, Hyogo 658-0082 Japan; ^4^ Current contact address: Department of Chemistry Faculty of Science Hokkaido University Sapporo Hokkaido 060-0810 Japan; ^5^ Chemistry Department Durham University South Road Durham DH1 3LE UK; ^6^ Department of Energy Conversion and Storage Technical University of Denmark 2800 Kongens Lyngby Denmark

**Keywords:** charge transfer, donor–acceptor, fluorescence, organic light-emitting diodes, triplet–triplet annihilation

## Abstract

Novel electron donor–acceptor–donor (D‐A‐D) compounds comprising dibenzo[*a,j*]phenazine as the central acceptor core and two 7‐membered diarylamines (iminodibenzyl and iminostilbene) as the donors have been designed and synthesized. Investigation of their physicochemical properties revealed the impact of C_2_ insertion into well‐known carbazole electron donors on the properties of previously reported twisted dibenzo[*a,j*]phenazine‐core D‐A‐D triads. Slight structural modification caused a drastic change in conformational preference, allowing unique photophysical behavior of dual emission derived from room‐temperature phosphorescence and triplet–triplet annihilation. Furthermore, electrochemical analysis suggested sigma‐dimer formation and electrochemical polymerization on the electrode. Quantum chemical calculations also rationalized the experimental results.

## Graphical abstract

**Plus 2**: C_2_ insertion into well‐known electron‐donating carbazole unit brought about a drastic change in the physicochemical properties of dibenzo[*a,j*]phenazine‐core twisted donor–acceptor–donor triad compounds. The slight structural modification at the donor unit led to a thermodynamic preference for quasi‐axial conformations, dual emission comprising room‐temperature phosphorescence and triplet–triplet annihilation, and unique electrochemical behavior.

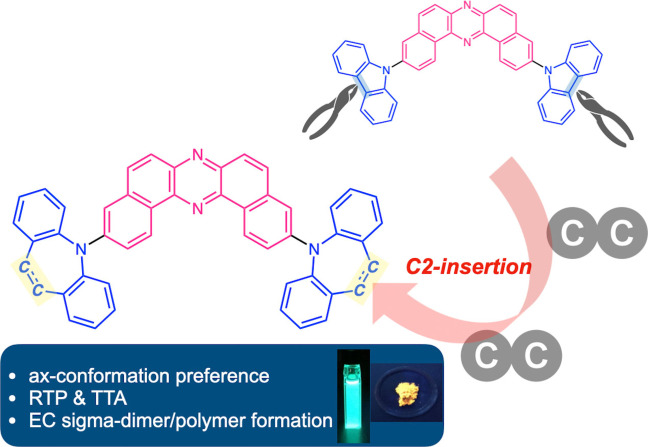

## Introduction

The carbazole (Cz) structure is a ubiquitous motif in natural products and pharmaceuticals.^[1]^ Not only that, the electron‐abundant aromatic component finds many applications as optoelectronic materials, such as hole‐transporting materials and organic semiconductors.^[2]^ Also, the carbazole unit has emerged as the electron‐donors in twisted donor–acceptor (D‐A) conjugated thermally activated delayed fluorescence (TADF) materials.^[3]^ The planar and rigid structure of the Cz in the D‐A scaffolds allow twisting around the connecting C−N bond, leading to a reduced singlet‐triplet energy gap (Δ*E*_ST_) in the excited state. In stark contrast to Cz, iminodibenzyl (IDB) and iminostilbene (ISB), which are C_2_‐inserted homologues of Cz,^[4]^ have been much less explored as electron donors in D‐A twisted type organic functional materials.[^5^, ^6^, ^7^] In 2018, Dias and Grazulevicius reported that the attachment of ISB and IDB donors to quinoxaline acceptor unit allows unique mechanochromic luminescence properties and dual TADF and room‐temperature phosphorescence (RTP).^[5]^ Independently, Bryce and Monkman investigated the influence of introducing ISB donor onto the 9,9‐dimethylthioxanthene‐*S,S*‐dioxide (TXO2) acceptor unit on the structure and TADF properties of the D‐A‐D compounds in comparison with other analogous donors, thus suggesting the importance of conformations in blue TADF.^[6]^ They showcased that IDB donor lies between diphenylamine and dihydroacridine donors in terms of electron‐donating ability and conformational flexibility. And recently, Kaji and coworkers disclosed the utilization of IDB in through‐space charge‐transfer (TSCT) TADF^[7]^ material which is featured with very efficient reverse intersystem crossing (rISC).^[8]^ Nevertheless, any other systematic studies of the influence of the C_2_ insertion into the Cz unit on the physicochemical properties of D‐A type compounds have not been clarified.

We have designed and synthesized two dibenzo[*a,j*]phenazine (DBPHZ)‐cored D‐A‐D triads with IDB and ISB as the electron donors to probe the influence of installing the 7‐membered diarylamines in place of carbazoles on the properties.

## Results and Discussion

### Design and synthesis

**Figure 1:**
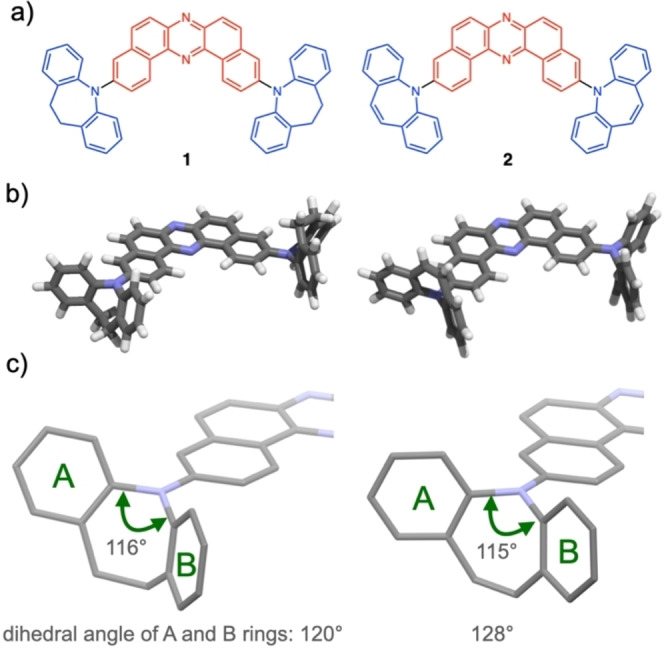
a) Chemical structures and b) the most thermodynamically stable conformers (ax–ax conformers) of **1** and **2**. c) Enlarged half part of ax–ax conformers of **1** and **2**.

Two donor–acceptor–donor (D‐A‐D) triads **1** and **2** were designed (Figure 1a), both of which have 7‐membered aromatic amine electron donors (i. e., IDB and ISB). Theoretical calculation suggested that ax–ax‐type conformers are much more thermodynamically stable (Figure 1b) than ax‐eq/eq–eq type conformers by about 5/11 and 7/15 kcal/mol for **1** and **2**, respectively. In both ax–ax conformers of **1** and **2**, the bent donors (dihedral angle of A and B for **1** and **2** ring is 120° and 128°, respectively) take the orientation so that the donor carbons directly connected to the nitrogen atom are almost on the same plane of DBPHZ acceptor (Figure 1c). Several conformers of each type were found, but their energies do not differ significantly within each type. The relative stability was not much affected by including a solvent model (for detailed calculations, see the Supporting Information).

**Scheme 1:**
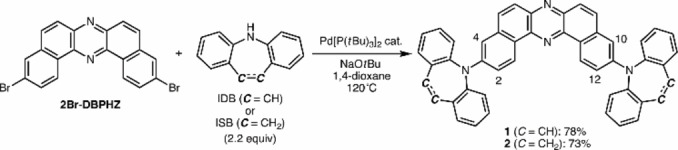
Synthesis of **1** and **2**.

Both D‐A‐D compounds **1** and **2** were readily synthesized through a Pd‐catalyzed Buchwald‐Hartwig double amination of 3,11‐dibromo‐dibenzo[*a,j*]phenazine (2Br‐DBPHZ)^[9]^ with IDB or ISB in good yields, respectively (Scheme 1; for detailed experimental procedures, see the Supporting Information). Both compounds were obtained as bright yellow powdery solids after re‐precipitation from CHCl_3_/*n*‐hexane solution. Both compounds were suitably characterized by common spectroscopy (i. e., ^1^H and ^13^C NMR, IR, and MS; for the detailed data, see the Supporting Information). It is noted that the signals corresponding to ^1^H nuclei at the 2 (12) and 4 (10) positions are observed at the upfield regime in ^1^H NMR spectra (6.6–7.1 ppm; for detailed spectra, see the Supporting Information), when compared to the D‐A‐D analogues having bis(*tert*‐butyl)carbazole (*t*‐BuCz) and diphenylamine (DPA) donors (>7.5 ppm).[^10^, ^11^] This distinct upfield shift would support the preference of ax–ax conformers in **1** and **2** in solutions, where the lone pair of the nitrogen atom in the donors effectively conjugate with the acceptor to increase the electron density at the 2 and 4 positions. An alternate possibility of such an upfield shift can be rationalized by the effect of the anisotropic ring current from the donors in the ax–ax conformers.

### Steady‐state photophysical properties

**Figure 2:**
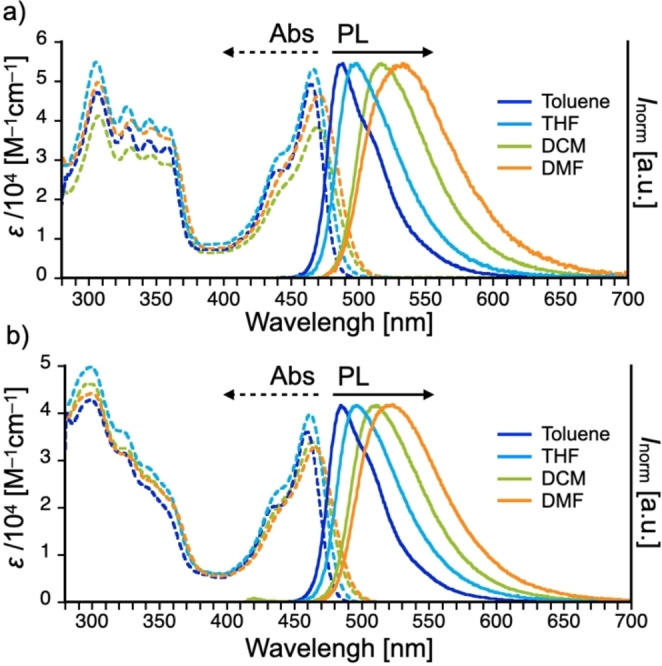
UV‐vis absorption and PL spectra of diluted solutions of a) **1** and b) **2** in various organic solvents (*c*=10^−5^ M, *λ*_ex_=400 nm).

Initially, the steady‐state photophysical properties of **1** and **2** in diluted solutions (*c*∼10^−5^ M) were investigated (Figure 2; a summary of the data is given in Table 1). The D‐A‐D compound with two IDB donors **1** exhibited absorptions with large molar absorption coefficient (*ϵ*) in the two regions: one with π–π* transitions with vibronic structures between 300–370 nm and the ones with hybrid transitions of intramolecular charge‐transfer (ICT) and π–π* character between 420–500 nm (dotted lines in Figure 2a). The mixing of CT and π‐π* character in the absorption in the lower‐energy region is evident from the slight red‐shift of the onset of the longest wavelength as a function of solvent polarity (dotted lines in Figure 2a) and indicated by the theoretical calculations (see below). The absorption maxima wavelength of **1** (*λ*_abs_=467 nm) and **2** (*λ*_abs_=462 nm) in THF (Table 1) lie in the middle of C_2_‐bridge‐free D‐A‐D analogues (with *t*‐BuCz: *λ*_abs_=449 nm;^[10]^ with DPA: *λ*_abs_=474 nm),^[11]^ indicating the C_2_‐insertion allows tuning optical bandgap as the results of balancing the twisting angle around the D−A bond and effective conjugation length. Reflecting the hybrid CT nature, the solution of **1** showed moderate positive photo‐luminochromism as the function of solvent polarity (solid lines in Figure 2a). As a whole, the iminostilbene counterpart **2** showed similar absorption profiles with those of **1**, but it is interesting to note that **2** showed less vibronic absorptions in the higher‐energy region (280–380 nm) and smaller *ϵ* in the lower energy absorption (420–500 nm) when compared to those of **1** (Figure 2b). The D‐A‐D compound **2** showed slightly blue‐shifted and narrower photoluminescence (PL) than those of **1**, thus implying the molecule experiences less structural relaxation in the excited state, probably due to the existence of a more rigid C=C bridge between the two phenyl groups in the donor. When compared with the emission peak of the *t*BuCz (*λ*_em_=537 nm)^[10]^ and the DPA (*λ*_em_=512 nm)^[11]^ analogues, the emission peaks of **1** and **2** appear in the bluer region, supporting the effect of C_2_‐insertion on rigidifying the structure in the excited states than *t*BuCz and DPZ analogues.

**Table 1:** Summary of the steady‐state photophysical properties of **1** and **2**.

	In solution^[a]^					Solid^[b]^	
	Solvent	*λ*_abs_ [nm]^[c]^	*ϵ* [M^–1^ cm^–1^]	*λ*_em_ [nm]^[d]^	*Φ*_PL_^[e]^	*λ*_em_ [nm]^[d]^	*Φ*_PL_^[e]^
**1**	toluene	465	48 500	489	0.54	596	0.09
	THF	467	52 900	498	0.49		
	CH_2_Cl2	470	39 700	517	0.49		
	DMF	471	47 900	534	0.50		
**2**	toluene	460	35 500	485	0.49	548	0.04
	THF	462	39 500	495	0.48		
	CH_2_Cl_2_	463	32 500	512	0.45		
	DMF	466	34 300	520	0.50		

[a] *c*=10^−5^ M. [b] The solid samples were prepared by reprecipitation from *n*‐hexane/CH_2_Cl_2_. [c] The longest absorption maxima. [d] *λ*_ex_=400 nm. [e] The photoluminescence quantum yields (PLQYs) were determined by integral sphere.

The reprecipitation of the D‐A‐D compounds from *n‐*hexane/CH_2_Cl_2_ solutions gave bright yellow powdery solids in both cases. Upon the irradiation with the UV lamp, these solids exhibited yellowish‐orange photoluminescence (PL; *λ*_em_ 596 nm for **1**; *λ*_em_ 548 nm for **2**). Different from the PL spectra of solutions, the steady‐state PL spectra of the solids (powder and neat thin film) of these compounds appears quite different (Figure S1 in the Supporting Information), thus indicating the operation of strong intermolecular interactions among the molecules and inhomogeneity of conformations in the aggregate states. It is noted that compound **2** displayed distinct morphology‐dependent emission behavior, suggesting that conformations would also be affected by the process of fabrication of materials.

### Electrochemical properties

**Figure 3:**
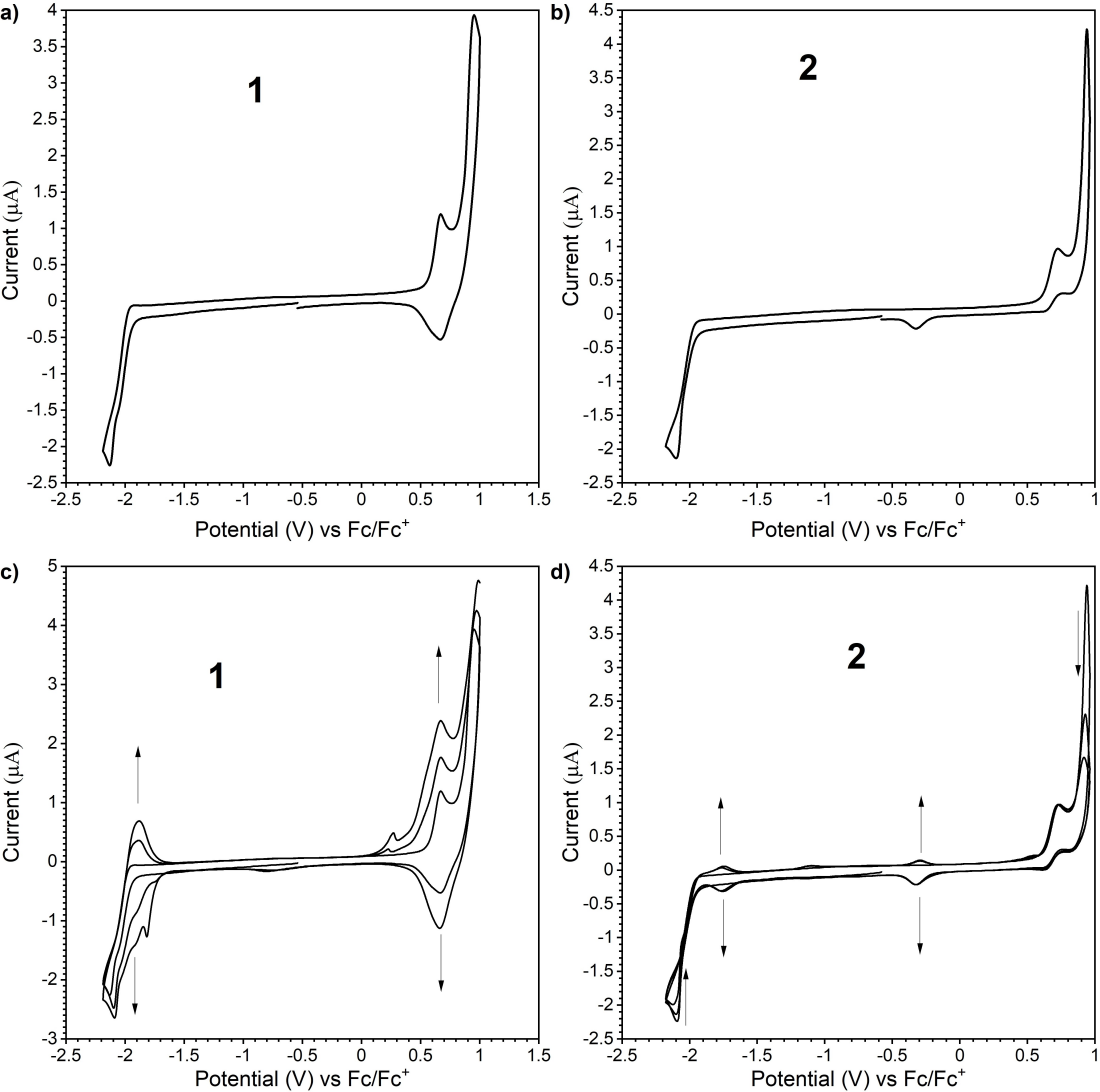
Cyclic voltammetry of a single scan of a) **1** and b) **2**. Multiple scans of c) **1** and d) **2** (*c*=1 mM) in CH_2_Cl_2_ containing Bu_4_NBF_4_ (0.1 M) electrolyte.

**Figure 4:**
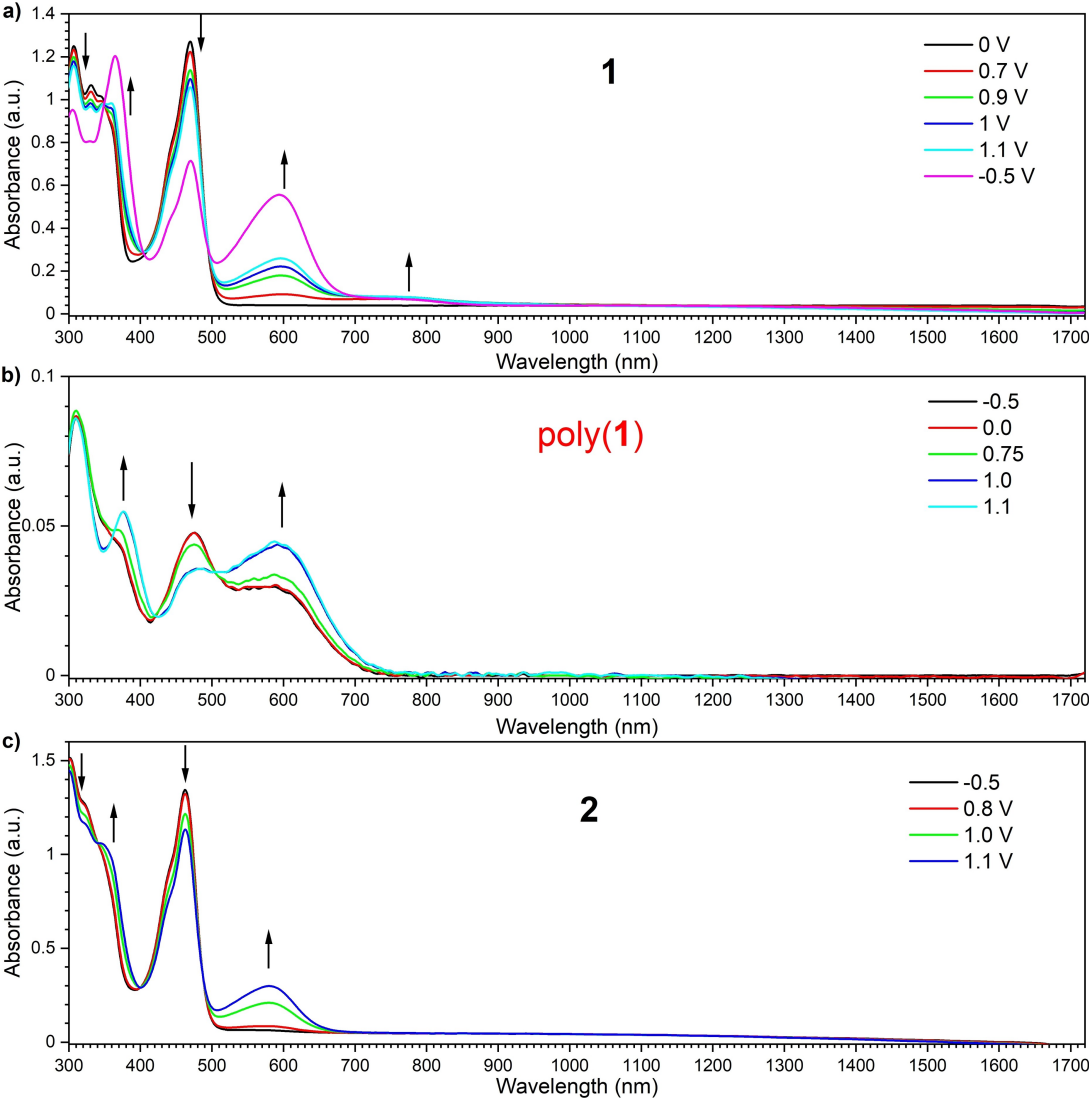
UV‐vis‐NIR spectro‐electrochemistry of a) **1** (*c*=1 mM), b) poly‐**1**, synthesized under the conditions presented in Figure S2d, and c) **2** in CH_2_Cl_2_ with Bu_4_NBF_4_ (0.1 M) electrolyte.

Electrochemical and spectro‐electrochemical studies of materials **1** and **2** showed remarkably different responses, and their responses were analyzed (Figures 3 and 4, below). Cyclic voltammetry (CV) in both cases showed two‐step oxidation with two irreversible redox processes (Figure 3a and b), but these processes were completely different. In the case of **1**, two oxidation peaks (^ox1^ *E*=+0.67 V and ^ox2^ *E*=+0.95 V) and one reduction peak (^red^ *E*=−2.13 V) were observed (Figure 3a). During the oxidation, we observed i) successive rise of the peak current with repeated scans and ii) the shift of oxidation onset to the lower potentials (Figure 3c). The first aspect suggests that conductive species are deposited on the electrode with increased active areas. The second aspect indicates the extension of the conjugation length of the active materials through electrochemical oxidation. Taken together, the observed effect could suggest the deposition of conjugated polymers of **1** on the electrode. According to the follow‐up analysis, it turned out the electropolymerization starts at ^ox1^ *E* potential, and it boosts when the switching potential approaches the ^ox2^ *E* value (Figure S2). Interestingly, in either case, similar conjugated polymers with good electrochemical stability were obtained (Figure S2). From the onset potentials of the first oxidation and reduction, the HOMO and LUMO energy levels of **1** and the resulting polymer were determined to be −5.65/−3.14 eV and −5.38/−3.31 eV, respectively.

As for the D‐A‐D compound with ISB donors **2**, two oxidation peaks at ^ox1^ *E*=+0.73 V and ^ox2^ *E*=+0.94 V, and one reduction peak at ^red^ *E*=−2.10 V were observed (Figure 3b). When compared to compound **1**, the peak current is lower (Figure 3a vs. 3b). This would be related to the decrease in the active area of the electrode, probably due to the deposition of low conductive species. The hint to answer what is causing the effect lies in the two newly observed redox couples arising at around −0.31 and −1.74 V (Figure 3d). Such an effect is usually caused by the formation of sigma dimers of the compound on the electrode.^[12]^ Sigma dimers affect the conjugation of the compound and decrease the conductivity of the material. What is interesting is that the sigma‐dimer formation appears only when the process is carried out up to the second oxidation potential (Figure S3). From the onset potentials of the first oxidation and reduction of **2**, the HOMO and LUMO energy levels were determined to be −5.72 and −3.18 eV, respectively. The difference in the reactivity of **1** and **2** could be ascribed to the presence of the C=C bridging unit in the donor unit, which might allow for the delocalization of (di)radical (di)cation generated through the electrochemical process to stabilize. The HOMO energy values of **1** and **2** are much closer to that of DPA analogue (−5.59 eV)^[11]^ than that of *t*BuCz (−5.79 eV) analogue,^[10]^ probably due to conformational consequence. This is understandable by the fact that the LUMO are less affected by the difference of D unit (the LUMO energies of *t*BuCz analogue: −3.37 eV;^[10]^ the LUMO energies of DPA analogue: −3.29 eV^[11]^).

The additional spectro‐electrochemical analysis of the potentiostatic oxidation and reduction process jointly with the electron paramagnetic resonance (EPR) spectrometer was conducted to analyze the formation of charge carrier species (Figure S4). The obtained results proved that in both cases (i. e., **1** and **2**), polarons are formed during the oxidation and reduction processes (Figure S4). The intensities of the EPR spectra are very low during the oxidation, whereas the signals were quite intense during the reduction process (Figure S4b and d). Moreover, during the reduction, the resolved spectra were acquired, which implies that the spin is localized around the nitrogen atoms on the acceptor unit, which is consistent with the calculated spin density (Figures S6 and S7).

UV‐vis‐NIR spectro‐electrochemical analysis of compound **1** shows that new strongly vibronic bands between 500–700 and 700–900 nm arose, while the absorption band ascribed to the neutral species decreased upon application of a positive potential (Figure 4a). The newly observed band peaked at around *λ*=600 nm is attributed to the formation of a radical cation species, and the band seen between 700–800 nm indicates the formation of radical cation on poly‐**1** (Figure 4b), not on a monomer. Any bipolaronic bands formation was not observed for both compounds. UV‐vis‐NIR spectro‐electrochemistry of **2** during oxidation shows the formation of a single broad band at around *λ*_max_=580 nm, which indicates the generation of radical cation species **2** ^.+^ (Figure 4c).

### Photoluminescence in matrices

**Figure 5:**
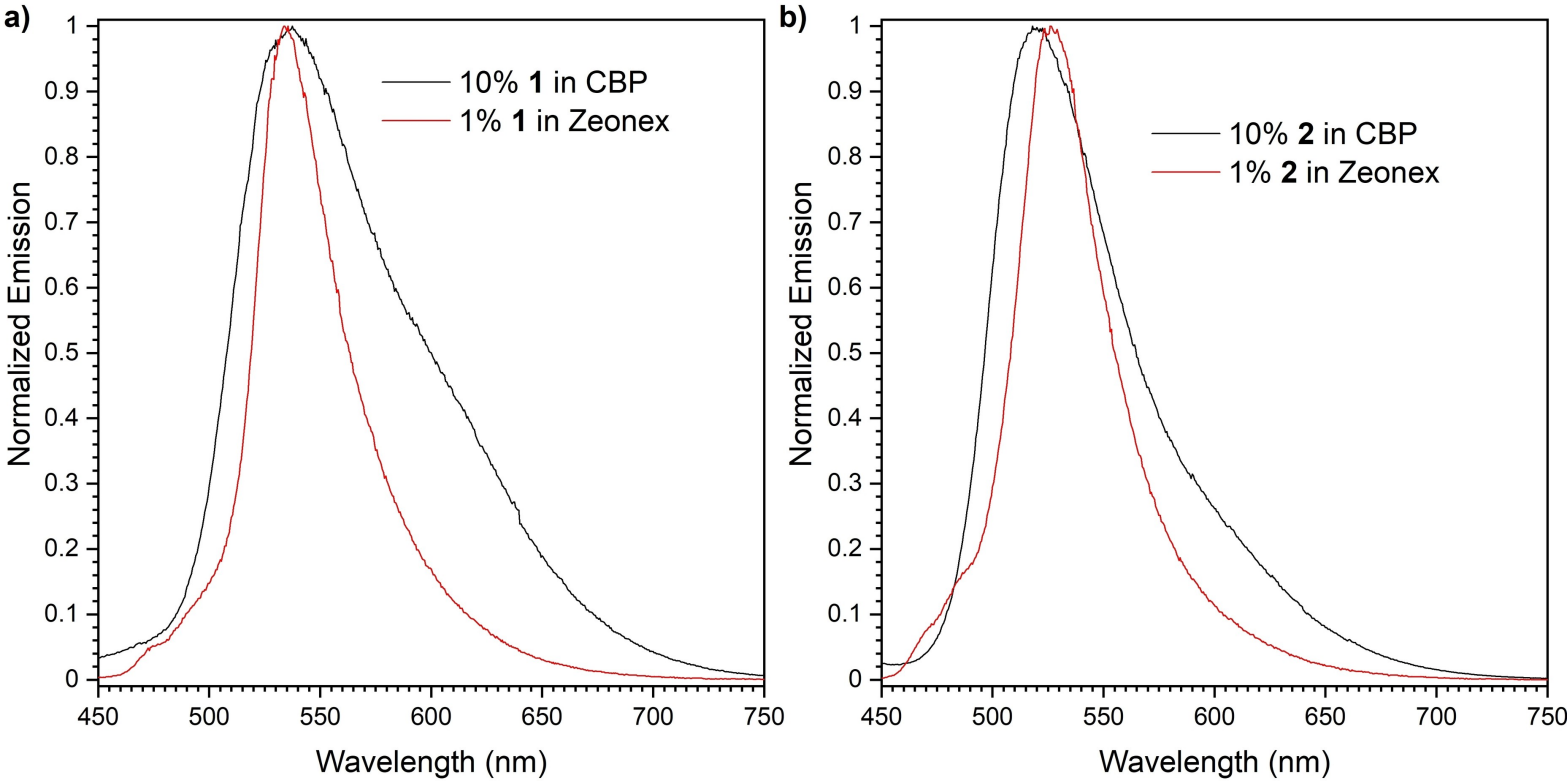
PL spectra of a) **1** and b) **2** in CBP and Zeonex® matrix (*λ*_ex_=355 nm).

The emissive properties of **1** and **2** in two matrices were investigated using a nonpolar polymer Zeonex® and CBP [4,4’‐bis(*N*‐carbazolyl)‐1,1’‐biphenyl]. CBP was chosen based on the HOMO‐LUMO properties of **1** and **2** for OLED applications. The photoluminescence (PL) of each compound in the Zeonex® matrix displayed a narrow single peak emission, suggesting that the PL is irradiated from the ^1^LE state. In contrast, broader emission spectra with a tail, which could be a sign of the emission from the ^1^CT excited state, was observed in the CBP matrix (Figure 5). Although spectra shape and half‐width are quite different in the two matrices, that much shift in the maximum peak wavelength was not observed (Figure 5). This could imply that there is a very small disturbance in vibronic levels of the compounds even in a more rigid CBP host (Figure 5).

### Time‐resolved spectroscopic analysis

**Figure 6:**
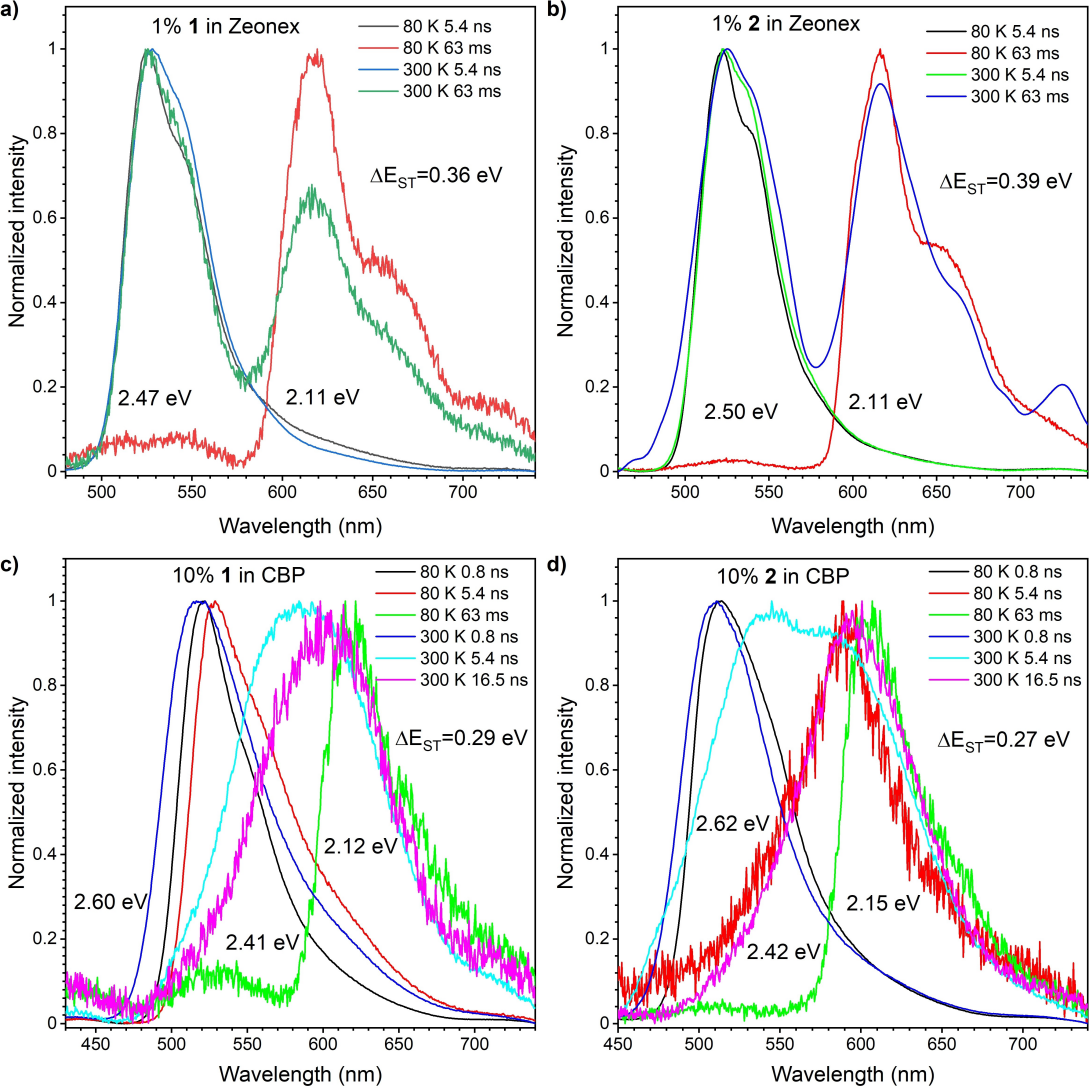
Normalized emission spectra of 1 wt% of a) **1** and b) **2** in Zeonex® and of 10 wt% of c) **1** and d) **2** in CBP at various delay times at 300 and 80 K. The singlet (S_1_) and triplet (T_1_) energies were estimated from the onset wavelength of respective emission spectrum.

**Figure 7:**
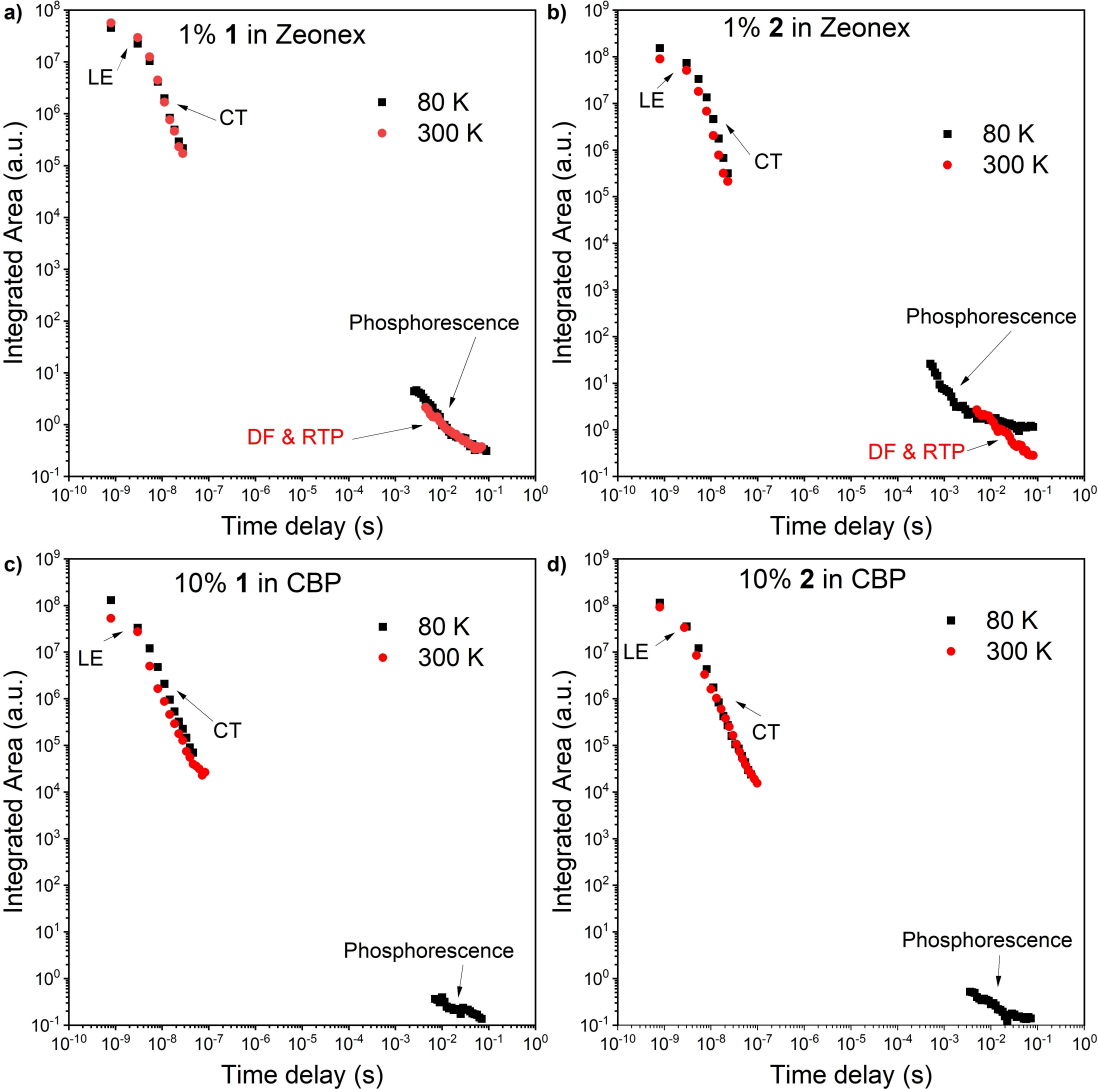
The emission intensity of **1** against delay time measured in a) Zeonex® and c) CBP at different temperatures. Emission intensity of **2** against delay time measured in b) Zeonex® and d) CBP at different temperatures.

Time‐resolved luminescence spectroscopy of the compounds in matrices revealed that the emission from both compounds decays within two distinct time regions. The first, decaying with a lifetime within the nanosecond time regime in all materials, is attributed to prompt emission (PE) from the singlet excited state (S_1_; Figures 6 and 7). There is a significant difference between the PE of the compounds in the Zeonex® and CBP matrix. In Zeonex® at room temperature, typical prompt fluorescence from the singlet localized excited state (^1^LE) was observed (Figure 6a and b). In stark contrast, in the CBP matrix, emissions firstly appeared from ^1^LE, but the emission from the charge‐transfer excited state (^1^CT) followed right after 3 ns delay and disappeared right after 16 ns (Figure 6c and d). In both cases, the lifetime of the emission from ^1^LE and ^1^CT is around 0.8 and 3.5 ns, respectively. Such fast emission behavior is not a usual effect, thus suggesting that the emission is irradiated from two different conformers with different dihedral angles between the two phenylene planes in the 7‐membered donor units, due to the flexible nature of IDB and ISB.

There is a significant difference at longer delay times. In the millisecond delay time region, delayed emission (DE) was observed (Figure 7). Depending upon the experimental temperature in Zeonex®, the DE from both the singlet and triplet excited states was observed on similar millisecond timescales. Therefore, the emission from each state is most easily elucidated upon spectral inspection at different temperatures (Figure 7). In Zeonex®, the long‐lived emission component observed at a low temperature (80 K) should be ascribed to phosphorescence (*E* ${}_{T{}_{1}}$ =2.11 eV for both compounds). This value is much lower than those of *t*BuCz analogue (*E* ${}_{T{}_{1}}$ =2.34 eV)^[10]^ and DPA analogue (*E* ${}_{T{}_{1}}$ =2.33 eV),^[11]^ indicating more effective conjugation length in matrix in consistent with ax–ax conformation. When the system is heated up, dual emission, consisting of the emission from the triplet state (i. e., RTP) and the delayed emission from the singlet state ^1^LE probably caused via triplet‐triplet annihilation (TTA) process,^[13]^ was observed (Figure 6a and b). The analysis of laser pulse fluence dependency of the delayed emission intensity integral (Figure S5) revealed that the slope is almost 2, which supports the TTA process. The emission started at a very long delay time (ca. 5 ms delay), and it went off after 70 ms (Figure 7a and b).

The change of the host to CBP significantly affected emissive behavior. Firstly, the emission from ^1^LE in Zeonex® shifted to a higher energy level by ca. 0.1 eV for both compounds (Figure 6c and d). Secondly, no delay (after microsecond delay) component was observed, and only typical phosphorescence at 80 K temperature was observed (Figures 6c, d and 7c, d). This is an unusual phenomenon, as there is no direct reverse intersystem crossing (rISC) effect from the lowest ^3^LE to ^1^CT state, even though the compounds in CBP have narrower Δ*E*_ST_ than in Zeonex®. This also proves the idea that both fast emissive components are related with two different molecular conformations and that there is no direct connection between the triplet state on the acceptor (^3^LE) and the ^1^CT. This behavior is quite in contrast to that of the Cz‐type D‐A‐D compound, where the two excited states with a different character (i. e., ^3^LE and ^1^CT) are connected within the same conformer, and moderate rISC can occur with a similar Δ*E*_ST_ value (0.43 eV in Zeonex® and 0.33 eV in CBP) to yield TADF in both Zeonex® and CBP matrices.^[11]^

### OLEDs fabrication and characterization

**Figure 8:**
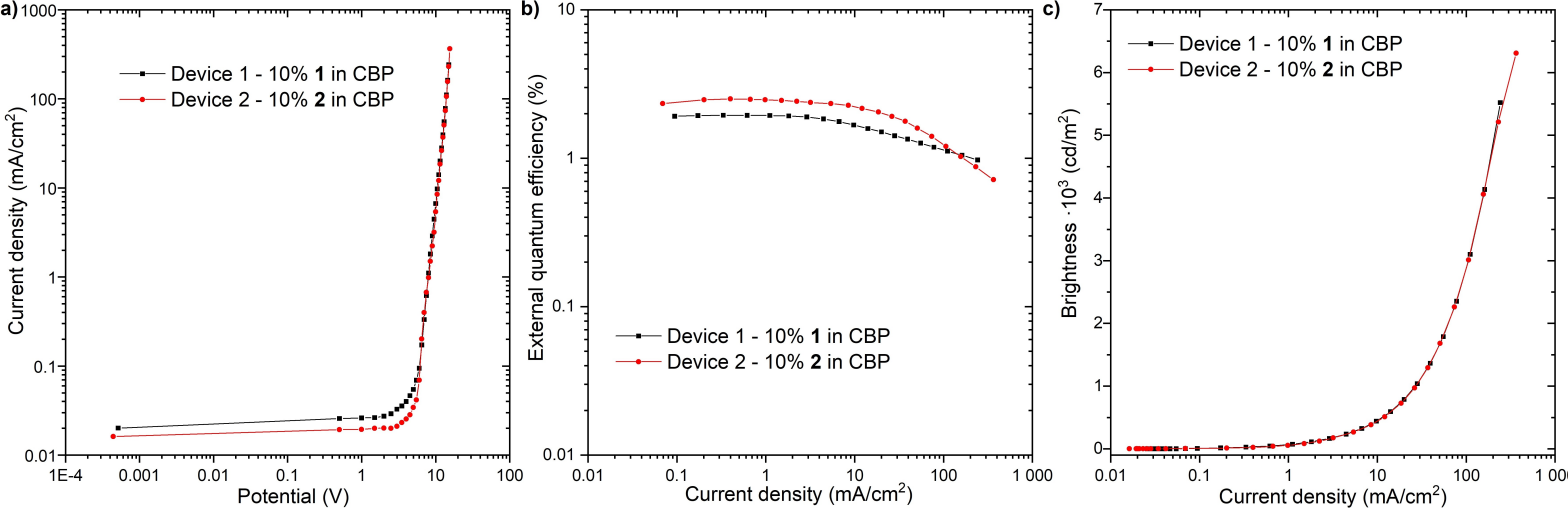
Device characteristics.

To apply compounds **1** and **2** to OLED application as an emitter, thermogravimetric analysis (TGA) was conducted. Both compounds showed high thermal stability under N_2_ gas [*T*_g_ (5 wt% loss) for **1**: 409 °C; *T*_g_ (5 wt% loss) for **1**: 440 °C] and air [*T*_g_ (5 wt% loss) for **1**: 409 °C; *T*_g_ (5 wt% loss) for **2**: 434 °C; Figure S6], indicating the feasibility of the fabrication of the emitting layer through vacuum thermal deposition. The OLED devices with the following configuration were fabricated: ITO/NPB [*N,N*’‐di(1‐naphthyl)‐*N,N’*‐diphenyl‐(1,1’‐biphenyl)‐4,4’‐diamine] (40 nm)/10 % **1** and **2** in CBP (20 nm)/TPBi [2,2’,2’’‐(1,3,5‐benzinetriyl)‐tris(1‐phenyl‐1‐*H*‐benzimidazole)] (50 nm)/LiF (1 nm)/Al (100 nm) (Figure 8). The characteristics of donor‐host OLED structures revealed a moderate external quantum efficiency (EQE) of 1.94 % for **1** and 2.5 % for **2** derivatives (Figure 8b). The luminance of the device based on **2** is also higher (6,300 cd/m^2^) than that with **1** (5,500 cd/m^2^) as the emitter. The only problem observed with the OLED based on **2** is the more intense roll‐off effect (Figure 8b) compared to the device with **1**. This might be caused by sigma‐dimer formation inside the layer presented in a different system before.^[14]^ The turn‐on voltage was found to be at around 6.0 V for both OLED devices, which is a slightly high value. Although the OLED configuration is different, the maximum EQEs of the OLEDs fabricated with *t*BuCz analogue (ca. 8 %)^[10]^ and DPA analogue (ca. 12 %)^[11]^ are much higher than those of **1** and **2**, as those emitters efficiently display TADF.

### Theoretical calculations

We performed electronic structure calculations for **1** and **2** to better understand their structure and electrochemical and photophysical properties. The computational details are included in the supporting information. Ground state calculations were done at the PBE0/cc‐pVDZ level and excited state calculations at the ω*PBE/cc‐pVDZ level, where ω* refers to the optimally tuned range separation parameter. The conformational search followed by geometry optimization at the density functional theory (DFT) level revealed that ax–ax conformations are thermodynamically the most stable (by 7 and 15 kcal/mol for **1** and **2**, respectively), accounting essentially for all the conformers present (Table S1). However, calculations for the charged molecules revealed that the energy gap between different conformers changes upon removal or addition of an electron (Table S2). While for singly charged molecules, the ax–ax conformation is still almost the only one present, for dications, the energy difference between ax–ax and eq–ax conformers is below 1 kcal/mol, so a significant presence of the latter is expected.

**Figure 9:**
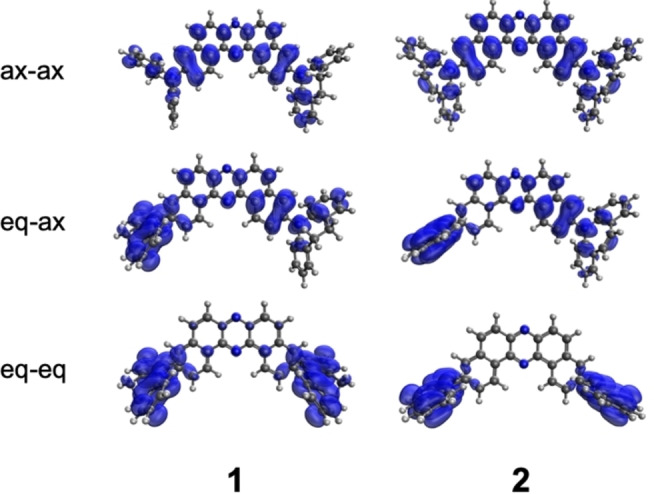
Spin density of diradical dication species of **1** and **2** in different conformers.

Interestingly, the behavior is different for **1** and **2** monocations, where the relative stability of eq–ax increases for **1** but decreases for **2**. This could explain why the changes in the CV occur already after the first oxidation for **1**, but only after the second for **2**. The calculated HOMO/LUMO levels are 5.32/2.43 eV for **1** and 5.36/2.42 eV for **2**, in reasonably good agreement with the experimental values (Table S3). The calculated second ionization potentials are 5.70 and 5.76 eV for **1** and **2**, respectively (Table S3). The resulting species is a diradical dication with the spin density delocalized over the entire molecule (Figures 9, S7 and S8).

The excited‐state calculations were done at the time‐dependent DFT level and included optimization of the S_1_ and T_1_ excited state geometries of representative ax–ax, eq–ax, and eq–eq conformers (Tables S4–S9). An implicit solvent model parametrized for toluene was used to account for the environmental effects. The calculated S_1_ absorption energies for ax–ax conformers are 2.94 and 2.97 eV, for **1** and **2**, respectively (Table S4). This overestimates the experimental values by 0.27 eV, but the slight shift between **1** and **2** is well reproduced. The calculations reveal that the geometry relaxation in the excited states leads to the decrease of the energy gap between eq and ax conformers (Table S5, S7, and S9), akin to the redox processes. The calculated emission energies are not directly comparable with the experiments measured in solid matrices but allow for the qualitative explanation of the spectra. For compound **1**, the S_1_ states of the eq–ax/eq–eq conformers are only 0.16/0.07 eV higher in energy than ax–ax (Table S7). In the polar CBP matrix, the differences would be even smaller, or the states would reverse their order, which explains the appearance of the delayed ^1^CT emission, due to the conformational change in the excited state. An analogous mechanism is expected for **2**, whose eq‐ax conformer is 0.22 eV higher in energy than ax–ax (Table S7), while the optimization of the eq–eq conformer failed. The calculated T_1_ states for the ax–ax conformers have 1.83/1.84 eV energies (Table S8), in good agreement with the experiment. The planarization of the donors in the T_1_ states can support low‐energy (1.1 eV) ^3^LE states localized on the donor unit, but these decay non‐radiatively and are not reflected in the spectra. The lack of efficient TADF in both molecules is evident from the large singlet‐triplet gaps calculated at the T_1_ equilibrium geometries, which are in the 0.66–0.78 eV range, depending on the conformer.

## Conclusion

We have synthesized dibenzo[*a,j*]phenazine‐cored donor–acceptor–donor (D‐A‐D) triads with C_2_ homologues of carbazole electron donors. Investigation of their physicochemical properties revealed the effect of replacing Cz with the conformationally more flexible 7‐membered aromatic amines (IDB and ISB). In contrast to the carbazole analogue, the D‐A‐D compounds disclosed here preferentially take ax–ax conformations over the eq‐type conformations. The C_2_ bridging of the Cz donors allowed a drastic change in photophysical behavior from moderate TADF to dual emission comprising of RTP and TTA from the single molecule. Moreover, electrochemical analyses uncovered the unique electropolymerization and dimerization phenomena of the D‐A‐D systems. Although the ISB and IDB donors might not be suitable for an efficient TADF emitter based on this acceptor system, the conformationally flexible nature of the donor and propensity to dimerize and polymerize under the action of electrochemical stimuli might open the way to developing stimuli‐responsive organic functional materials in the future.

## Conflict of interest

The authors declare no conflict of interest.

## Supporting information

As a service to our authors and readers, this journal provides supporting information supplied by the authors. Such materials are peer reviewed and may be re‐organized for online delivery, but are not copy‐edited or typeset. Technical support issues arising from supporting information (other than missing files) should be addressed to the authors.

Supporting Information
