# Supplementary material for: The Impact of C2 Insertion into a Carbazole Donor on the Physicochemical Properties of Dibenzo[a,j]phenazine‐Cored Donor–Acceptor–Donor Triads
Source: Chemistry. 2021 Aug 13;27(53):13390–8. doi: 10.1002/chem.202101654 (PMC8519004; doi:10.1002/chem.202101654)
Supplement: Supplementary file 1 — Supporting Information [file CHEM-27-13390-s001.pdf]

Chemistry–A European Journal

Supporting Information

The Impact of C_2 Insertion into a Carbazole Donor on the Physicochemical Properties of Dibenzo[*a,j*]phenazine-Cored Donor–Acceptor–Donor Triads

Paola Zimmermann Crocomo, Takahito Kaihara, Soki Kawaguchi., Patrycja Stachelek, Satoshi Minakata, Piotr de Silva,* Przemyslaw Data,* and Youhei Takeda*

Table of Contents

**General Remarks**	S2–S4
**Synthetic Procedures and Compound Data of 1 and 2**	S4–S5
**Steady-State PL Spectra in the Solid States**	S6
**Additional CV Experiments**	S7–S8
**EPR Experiments**	S9
**Laser Pulse Fluence Dependency of Delayed Demission Intensity Integral**	S10
**Thermogravimetric Analysis (TGA)**	S10
**Copies of NMR Charts**	S11–S12
**Theoretical Calculations**	S13–S18
**References**	S19

General Remarks. All reactions were carried out under an atmosphere of nitrogen unless otherwise noted. Products were purified by chromatography on silica gel BW-300 and Chromatorex NH (Fuji Silysia Chemical Ltd.). Analytical thin-layer chromatography (TLC) was performed on pre-coated silica gel glass plates (Wako silica gel 70 FM TLC plate and Fuji Silysia Chromatorex NH, 0.25 mm thickness). Compounds were visualized with a UV lamp. Melting points were determined on a Stanford Research Systems MPA100 OptiMelt Automated Melting Point System. All ^{1}H and ^{13}C spectra except were recorded on a JEOL JMTC-400/54/SS Spectrometer (^{1}H NMR, 400 MHz; ^{13}C NMR, 100 MHz) using tetramethylsilane as an internal standard (for ^{1}H and ^{13}C NMR). Infrared spectra were acquired on a SHIMADZU IRAffinity-1 FT-IR Spectrometer. Mass spectra and High-resolution mass spectra were obtained on a JEOL JMS-700 Mass Spectrometer. Steady-state UV-vis spectra were recorded on a Shimadzu UV-2550 spectrophotometer. Steady-state emission spectra were recorded on a HAMAMATSU C11347-01 spectrometer with an integrating sphere. Cyclic voltammetry (CV) was performed with the Biologic SP150 system. UV-Vis-NIR spectroscopy and spectroelectrochemistry were performed using Ocean Optics QE6500 and NIRQuest matrix spectrometers. In situ EPR spectroelectrochemical experiments were undertaken using a JES-FA 200 (JEOL) spectrometer. Thermogravimetric analysis (TGA) was performed with TG/DTA-7200 system (SII Nano Technology Inc.).

Materials. Dehydrated 1,4-dioxane was used as received. 3,11-Dibromo-dibenzo[*a,j*]phenazine [CAS No. 1620543-64-7] was synthesized according to the reported procedure.[S1] 10,11-Dihydro-5*H*-dibenzo[*b,f*]azepine (iminodibenzyl) and 5*H*-dibenzo[*b,f*]azepine (iminodibensile) were purchased from a commercial source and used as received. Solvents of fluorescence spectroscopic grade were purchased from Nacalai Tesque Inc. and Kanto Chemical Co., Inc. for measurement of UV-vis and emission spectra. Dichloromethane (CHROMASOLV®, 99.9%) and *n*-Bu_4NBF_4 (99%, dried) for CV were purchased from Sigma Aldrich.

Photophysics. Phosphorescence, prompt fluorescence (PF), and delayed fluorescence (DF) spectra and fluorescence decay curves were recorded using nanosecond gated luminescence and lifetime measurements (from 400 ps to 1 s) using either third harmonics of a high energy pulsed DPSS laser emitting at 355 nm (Q-Spark-A50). Emission was focused on a spectrograph and detected on a sensitive gated iCCD camera (Stanford Computer Optics) with sub-nanosecond resolution. Temperature photophysical measurements were conducted in the Janis CCS-450 closed-cycle helium cytostatic system.

Devices. OLEDs have been fabricated on pre-cleaned, patterned indium-tin-oxide (ITO) coated glass

substrates with a sheet resistance of 20 Ω/sq and ITO thickness of 100 nm. All small molecules and cathode layers were thermally evaporated in Kurt J. Lesker Spectros 150 evaporation system under pressure of 10^{-7} mbar without breaking the vacuum. The sizes of the pixels were 4 mm^2, 8 mm^2 and 16 mm^2. All organic evaporated compounds were purified by CreaPhys organic sublimation system. All materials were purified by temperature-gradient sublimation in a vacuum. The characteristics of the devices were recorded using a 10-inch integrating sphere (Labsphere) connected to a Source Meter Unit and Ocean Optics USB4000 spectrometer.

CBP - 4,4′-bis(*N*-carbazolyl)-1,1′-biphenyl (Sigma Aldrich), NPB - *N,N′*-Di-1-naphthyl-*N,N′*-diphenylbenzidine (TCI-Europe), TPBi - 2,2',2"-(1,3,5-Benzinetriyl)-tris(1-phenyl-1-H-benzimidazole) (LUMTEC), LiF (99.995%, Sigma Aldrich), and Aluminium wire (99.9995%, Alfa Aesar) were purchased from the companies indicated in each parentheses. All materials were purchased from Sigma Aldrich or Lumtec and were purified by temperature-gradient sublimation in a vacuum.

Electrochemistry. Electrochemical measurements were performed in 0.1 M Bu_4NBF_4 (99%, Sigma Aldrich, dried) in dichloromethane (CHROMASOLV®, 99.9% Sigma Aldrich). Solutions were purged with argon before measurement. Electrodes: working (Pt disc 1 mm of diameter), counter (Pt wire), reference (Ag/AgCl calibrated against ferrocene). All cyclic voltammetry measurements performed at room temperature with a scan rate of 50 mV s^{-1}. Ionization potential (IP) and electron affinity (EA) were calculated from oxidation (E_{ox}) and reduction (E_{red}) potentials, respectively, using following equations: IP = E_{ox} + 5.1, EA = E_{red} + 5.1.

Simulations. The computational protocol was consistent with several stages. In the first step, we performed a conformer search using the UFF force field as implemented in the AMS package.[S2] From the generated conformers, we selected a set of qualitatively different conformations, which were further optimized at the density functional theory (DFT) PBE0/cc-pVDZ level using the QChem package.[S3] The analysis of results revealed three qualitatively different conformers for each compound, differing by the orientation of the IDB or ISB units. Therefore, we selected three conformation, one of the ax-ax, eq-ax, and eq-eq type, for each compound. Simulations of electrochemical properties were done at the PBE0/cc-pVDZ level using the PCM solvent models parametrized for DCM. To this end, we optimized the geometries of charged species and calculated IPs and EAs as total energy differences. To perform the excited state calculations, we used the optimally tuned range separated functional ω*PBE. The ω parameter was optimized for the most stable conformers of **1,** and **2** and the values were 162 and 166 $bohr^{-1}$, respectively. For further, calculations we used the average value ω=164 $bohr^{-1}$. The time-dependent DFT calculations were done at the ground-state geometries as well as optimized

S1 and T1 geometries using a non-equilibrium PCM solvation model parametrized for toluene (ε=2.38, ε_∞=2.24) as implemented in QChem.

Synthetic Procedures for D–A–D Compounds

3,11-Bis(10,11-dihydro-5*H*-dibenzo[*b,f*]azepin-5-yl)dibenzo[*a,j*]phenazine (1)

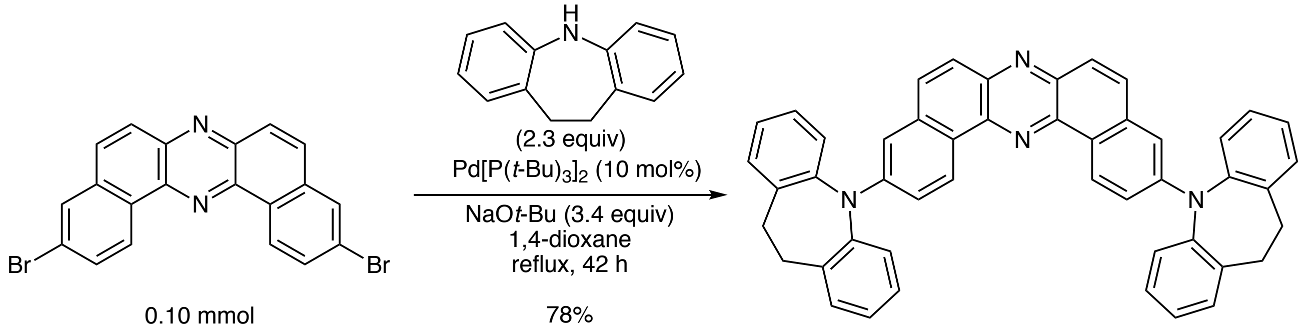

1

Two-necked reaction tube (10 mL) equipped with a stirring bar was charged with 3,11-dibromo-dibenzo[*a,j*]phenazine (43.8 mg, 0.10 mmol, 1.0 equiv) and 10,11-dihydro-5*H*-dibenzo[*b,f*]azepine (45.0 mg, 0.23 mmol, 2.3 equiv). The tube was transferred into a glovebox, and $Pd[P(^tBu_3)_2]$ (5.0 mg, 9.8 μmol, 10 mol%) and NaO*t*-Bu (33.0 mg, 0.34 mmol, 3.4 equiv) were added into the tube. The tube was sealed with two septum and taken out from the glovebox. To the tube, 1,4-dioxane (4 mL) was injected into the tube with a syringe through the septum. The resulting mixture was refluxed at a heated aluminum block (120 ˚C) for 42 h. The reaction mixture was allowed to cool to rt. Water (4 mL) was added, and the resulting mixture was extracted with $CHCl_3$ (10 mL × 3). The organic layer was combined, dried over Na_2SO_4, and filtered. Solvents were removed in vacuo to give the crude product, which was then purified on NH silica gel flash chromatography (eluent: *n*-hexane/$CHCl_3$ = from 99/1 to 9:1) to give product as yellow solid. The solid was further purified through re-precipitation from a *n*-hexane/$CHCl_3$ solution to give the product as yellow solid (52.3 mg, 0.078 mmol, 78%). mp >400 °C (dec.); T_d (5 wt% loss, under air) 409 °C; T_d (5 wt% loss, under N_2 gas) 409 °C; R_f 0.10 (on NH silica gel TLC, *n*-hexane/EtOAc 95:5); ^{1}H NMR (400 MHz, $CDCl_3$): δ 9.19 (d, J = 8.8 Hz, 2H), 7.83 (d, J = 9.2 Hz, 2H), 7.67 (d, J = 9.2 Hz, 2H), 7.53 (dd, J = 8.0, 2.0 Hz, 4H), 7.28–7.3 (m, 12H), 7.12 (dd, J = 8.8, 2.8 Hz, 2H), 6.96 (d, J = 2.8 Hz, 2H), 3.05 (s, 4H); ^{13}C NMR (100 MHz, $CDCl_3$): δ 149.1, 143.0, 141.1, 140.6, 138.1, 134.7, 131.2, 131.1, 129.8, 127.5, 127.3, 127.2, 126.1, 122.7, 114.4, 108.8, 30.7; IR (ATR, cm^{-1}): ν 3055, 1616, 1595, 1558, 1541, 1521, 1489, 1474, 1354, 1313, 1281, 1141, 999, 854, 789, 777. MS (FAB$^+$, NBA) *m/z* (relative intensity, %): 668 ([M+1+H]$^+$, 3), 667 ([M+H]$^+$, 6); HRMS (FAB$^+$, NBA:PEG + NaI): *m/z* calcd for $C_{48}H_{35}N_4$ (M+H) 667.2862, found 667.2865.

3,11-bis(5*H*-dibenzo[*b*,*f*]azepin-5-yl)dibenzo[*a*,*j*]phenazine (2)

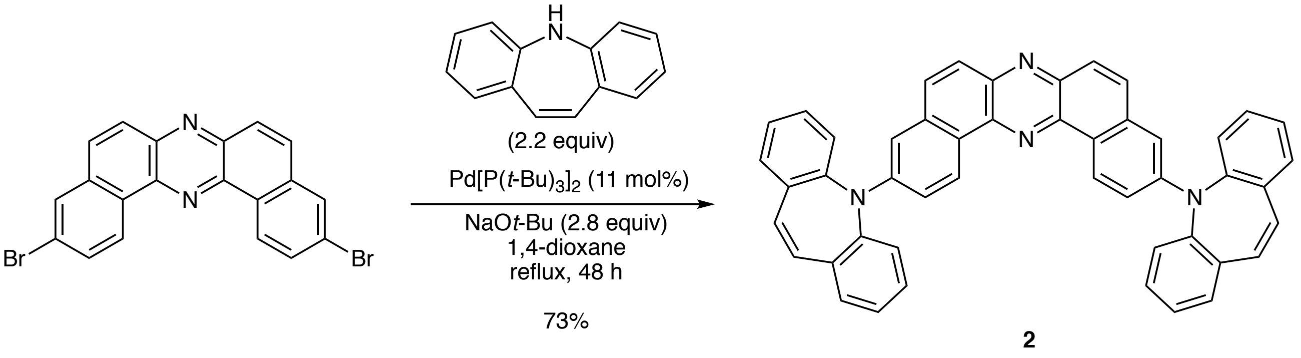

Two-necked reaction tube (10 mL) equipped with a stirring bar was charged with 3,11-dibromo-dibenzo[*a*,*j*]phenazine (43.8 mg, 0.10 mmol, 1.0 equiv) and 5*H*-dibenzo[*b*,*f*]azepine (43.0 mg, 0.22 mmol, 2.2 equiv). The tube was transferred into a glovebox, and Pd[P(^{t}Bu$_3$)$_2$] (5.7 mg, 11.1 μmol, 11 mol%) and NaO*t*-Bu (27.0 mg, 0.28 mmol, 2.8 equiv) were added into the tube. The tube was sealed with two septum and taken out from the glovebox. To the tube, 1,4-dioxane (1.5 mL) was injected into the tube with a syringe through the septum. The resulting mixture was refluxed at a heated aluminum block (120 ˚C) for 48 h. The reaction mixture was allowed to cool to rt. Water (4 mL) was added, and the resulting mixture was extracted with $CHCl_3$ (60 mL × 3). The organic layer was combined, washed with brine (50 mL), dried over Na_2SO_4, and filtered. Solvents were removed in vacuo to give the crude product as dark solids, which was then purified on NH silica gel flash chromatography (eluent: *n*-hexane/$CHCl_3$ = from 99/1 to 8:2) to give the product as yellow solid. The solid was further purified through re-precipitation from a *n*-hexane/$CHCl_3$ solution to give the product as yellow solid (48.7 mg, 0.073 mmol, 73%). mp >400 °C (dec.); T_d (5 wt% loss, under air) 434 °C; T_d (5 wt% loss, under N_2 gas) 440 °C; R_f 0.12 (on NH silica gel TLC plate, *n*-hexane/EtOAc 8:2); ^{1}H NMR (400 MHz, $CDCl_3$): δ 9.07 (d, J = 9.2 Hz, 2H), 7.78 (d, J = 9.2 Hz, 2H), 7.56–7.62 (m, 10H), 7.51 (dd, J = 7.2, 0.8 Hz, 4H), 7.42–7.46 (m, 4H), 6.87 (s, 4H), 6.79 (dd, J = 9.2, 2.8 Hz, 2H), 6.63 (d, J = 2.8 Hz, 2H); ^{13}C NMR (100 MHz, $CDCl_3$): δ 149.6, 142.5, 141.0, 140.6, 136.2, 134.3, 131.2, 130.57, 130.56, 130.1, 129.9, 127.4, 127.1, 125.7, 123.1, 113.7, 108.1; IR (ATR, cm^{-1}): ν 3057, 1616, 1591, 1541, 1474, 1435, 1356, 1304, 1283, 1260, 1148, 1032, 972, 854, 787, 741, 716. MS (FAB$^+$, NBA) *m*/*z* (relative intensity, %): 664 ([M+1+H]$^+$, 4), 663 ([M+H]$^+$, 7); HRMS (FAB$^+$, NBA:PEG + NaI): *m*/*z* calcd for $C_{48}H_{32}N_4$ (M+H) 663.2549, found 663.2544.

Steady-State PL Spectra in the Solid States

a)

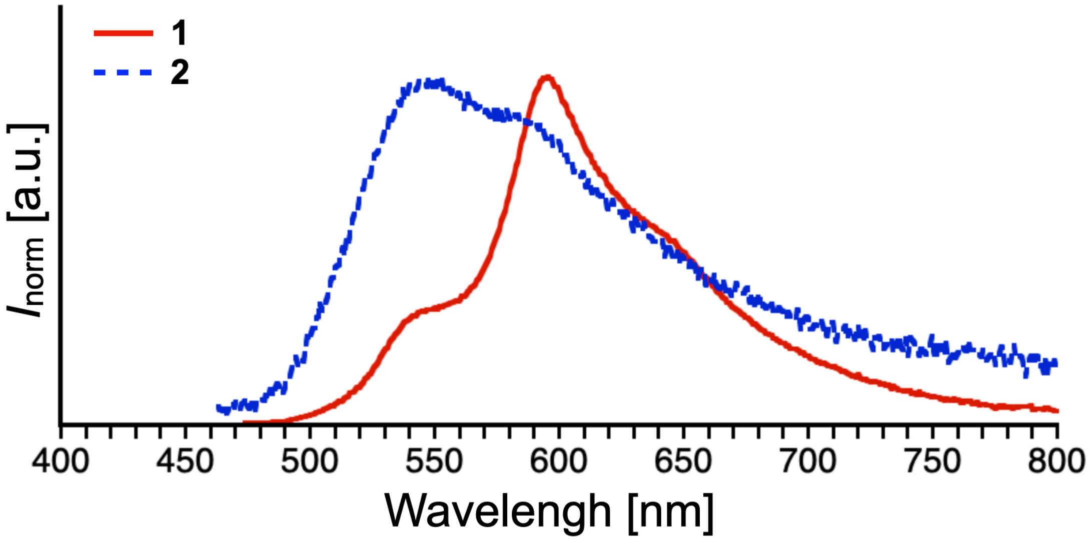

b)

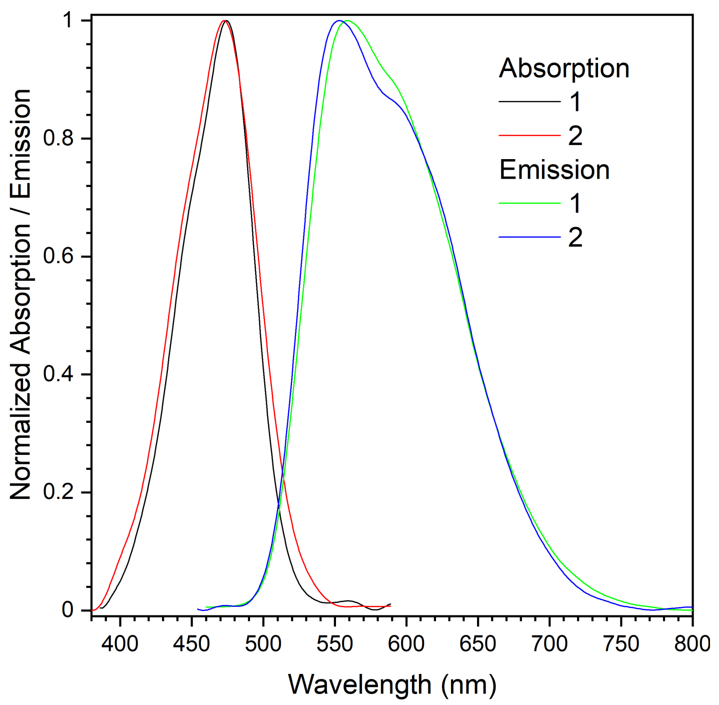

Figure S1. Steady-state PL spectra of **1** and **2** in a) powder and b) neat-thin film.

Additional CV Experiments

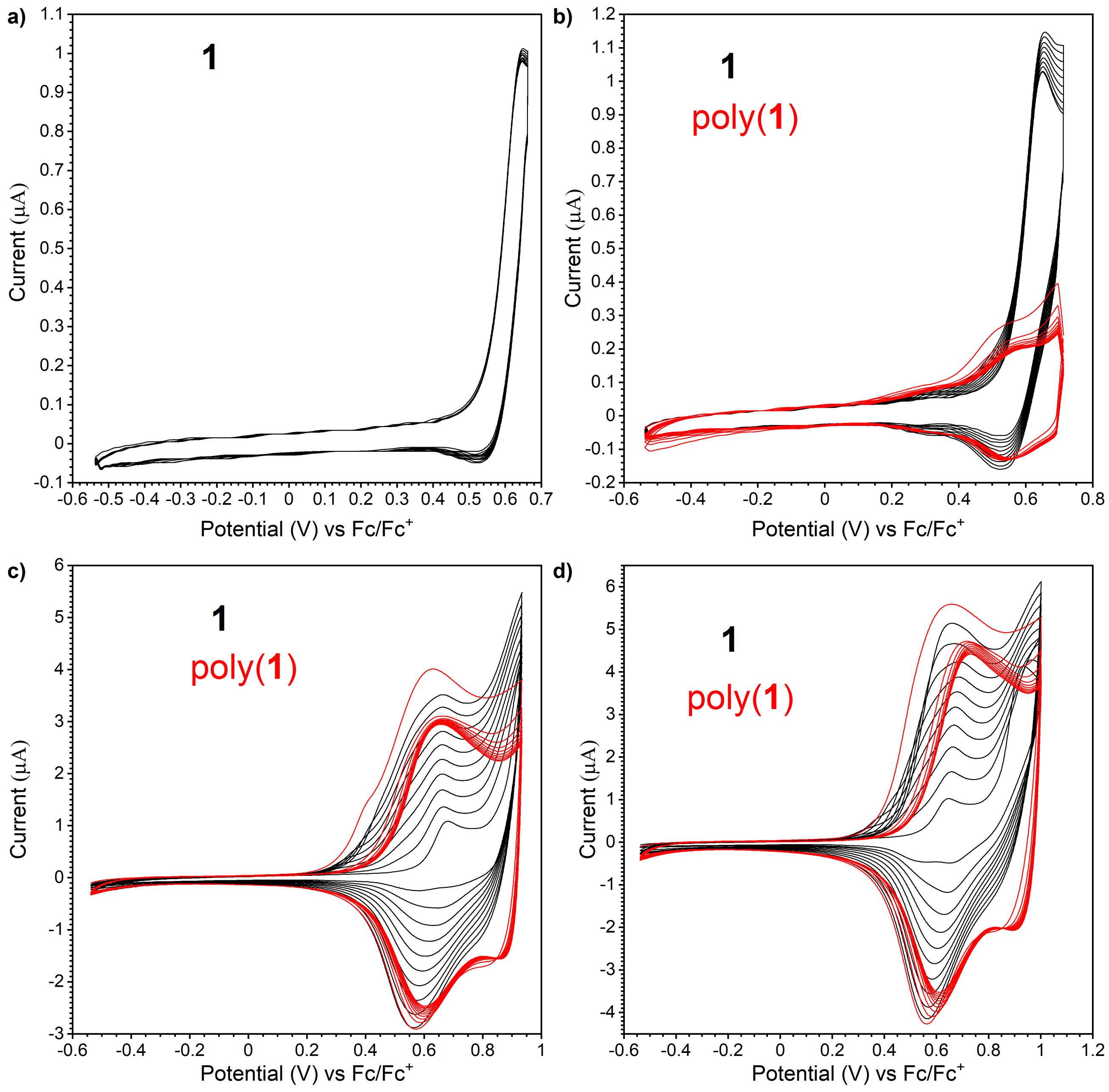

Figure S2. Cyclic voltammetry of **1** (*c* = 1 mM) in DCM Bu_4NBF_4 (0.1 M) electrolyte with different oxidation potential boundary condition. a) at the first peak, b) after the first peak, c) at the second peak, d) after the second peak. In the red, the electrochemical stability analysis of the obtained polymer.

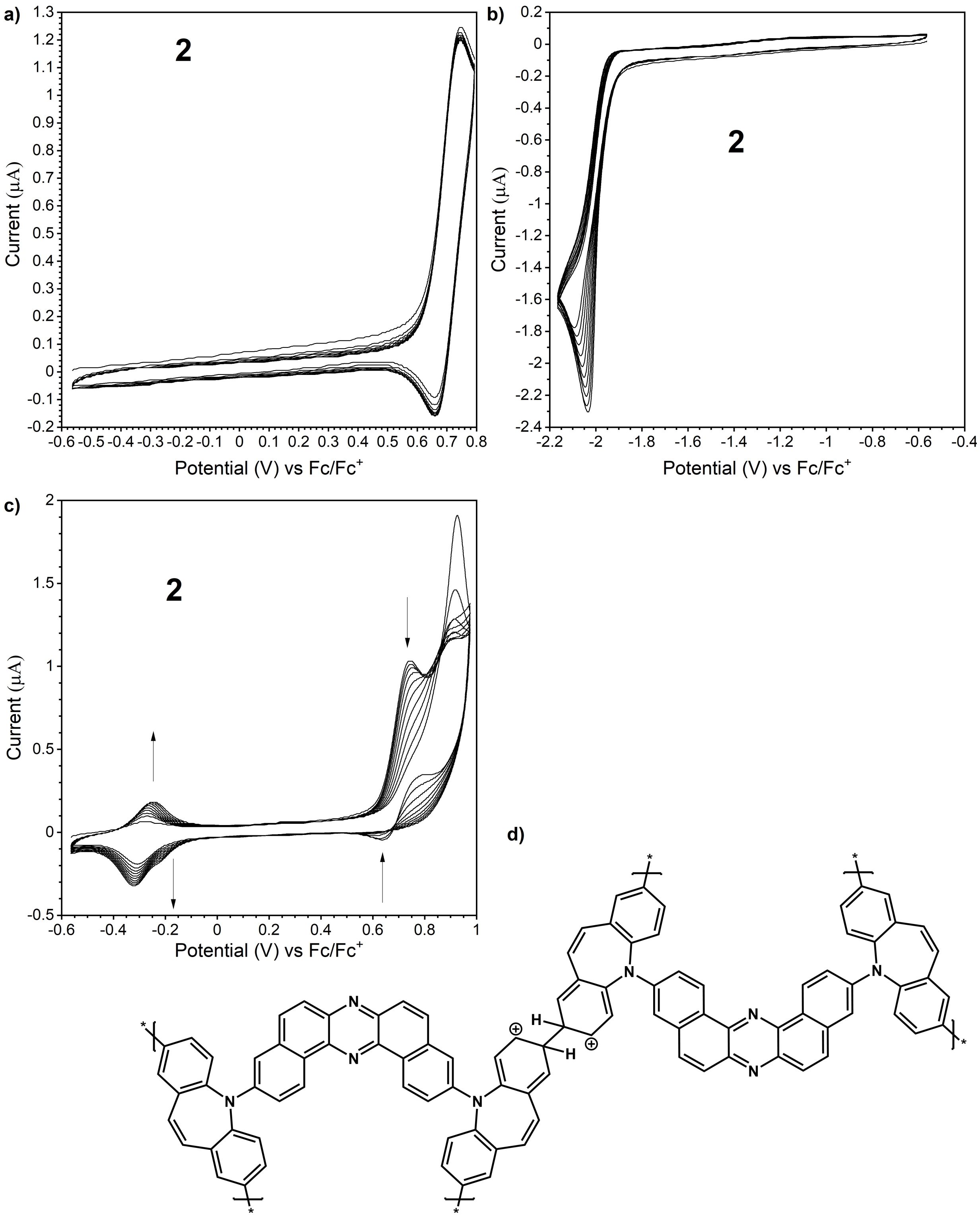

Figure S3. Cyclic voltammetry of **2** (c = 1 mM) in DCM Bu_4NBF_4 (0.1 M) electrolyte with different potential boundary condition. a) cyclic oxidation up to the first peak, b) cyclic reduction, c) cyclic oxidation up to the second oxidation peak, and d) a possible σ-dimer structure.

EPR Experiments

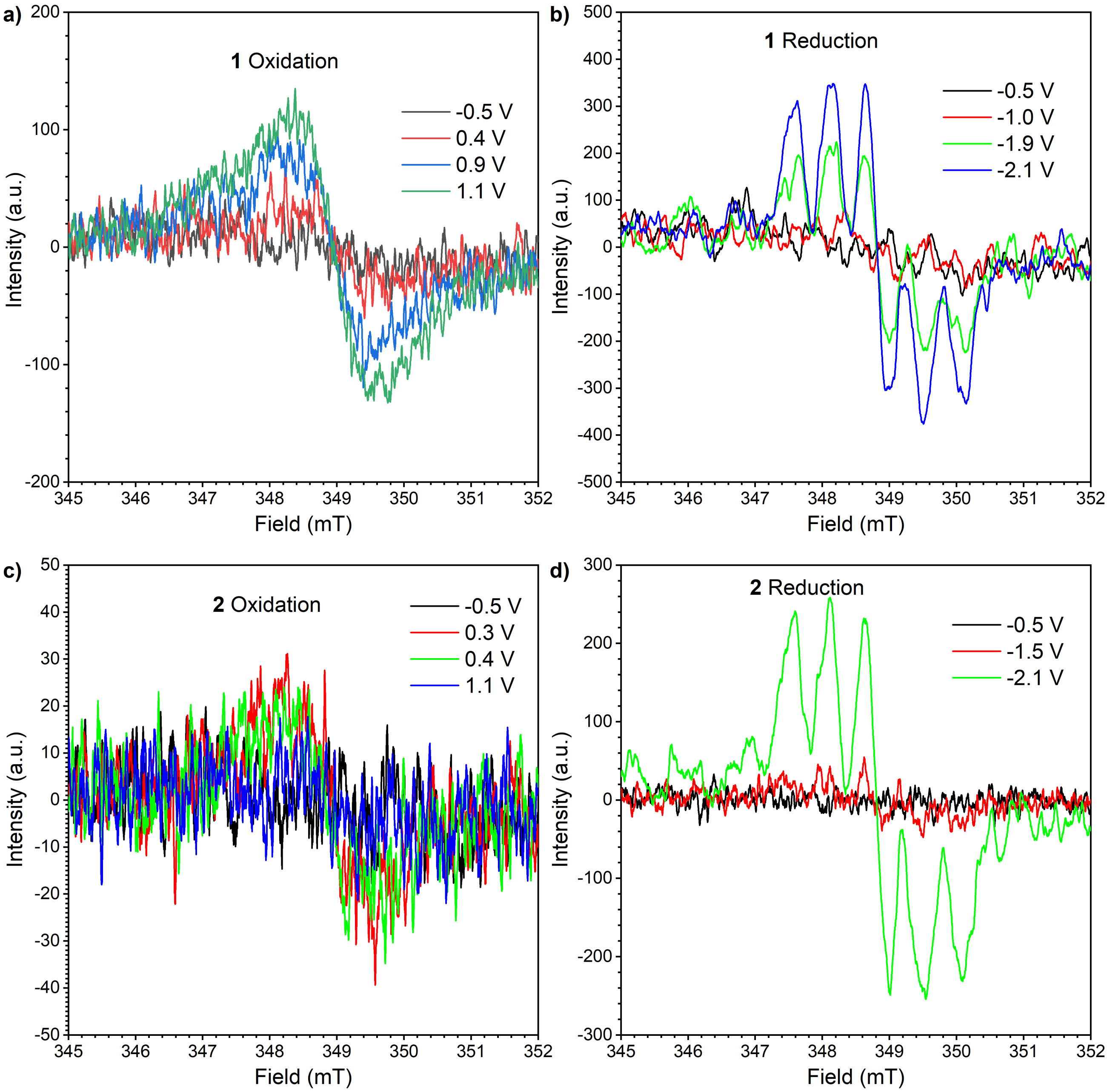

Figure S4. EPR spectra at potentiostatic oxidation and reduction processes of **1** and **2** (c = 1 mM) in DCM containing Bu_4NBF_4 (0.1 M) electrolyte.

Laser Pulse Fluence Dependency of Delayed Demission Intensity Integral

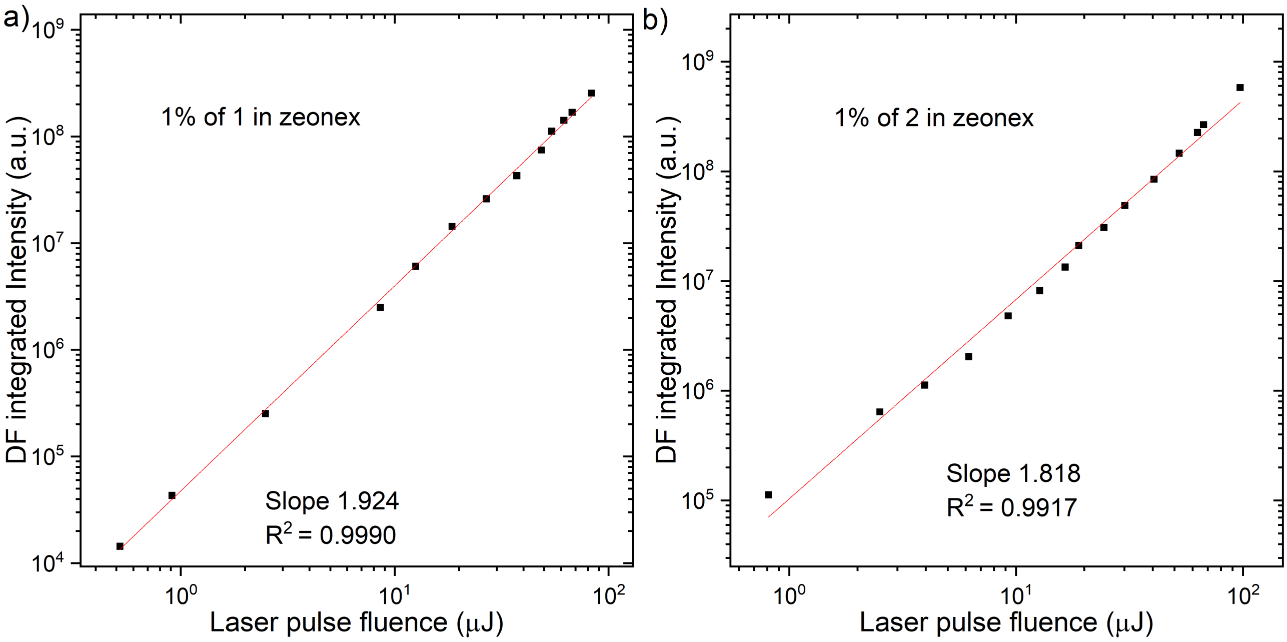

Figure S5. Integrated emission intensity *vs* laser pulse energy (μJ) of 1% a) **1** and b) **2** in Zeonex matrix at 300 K at delay time = 5 ms.

Thermogravimetric analysis (TGA)

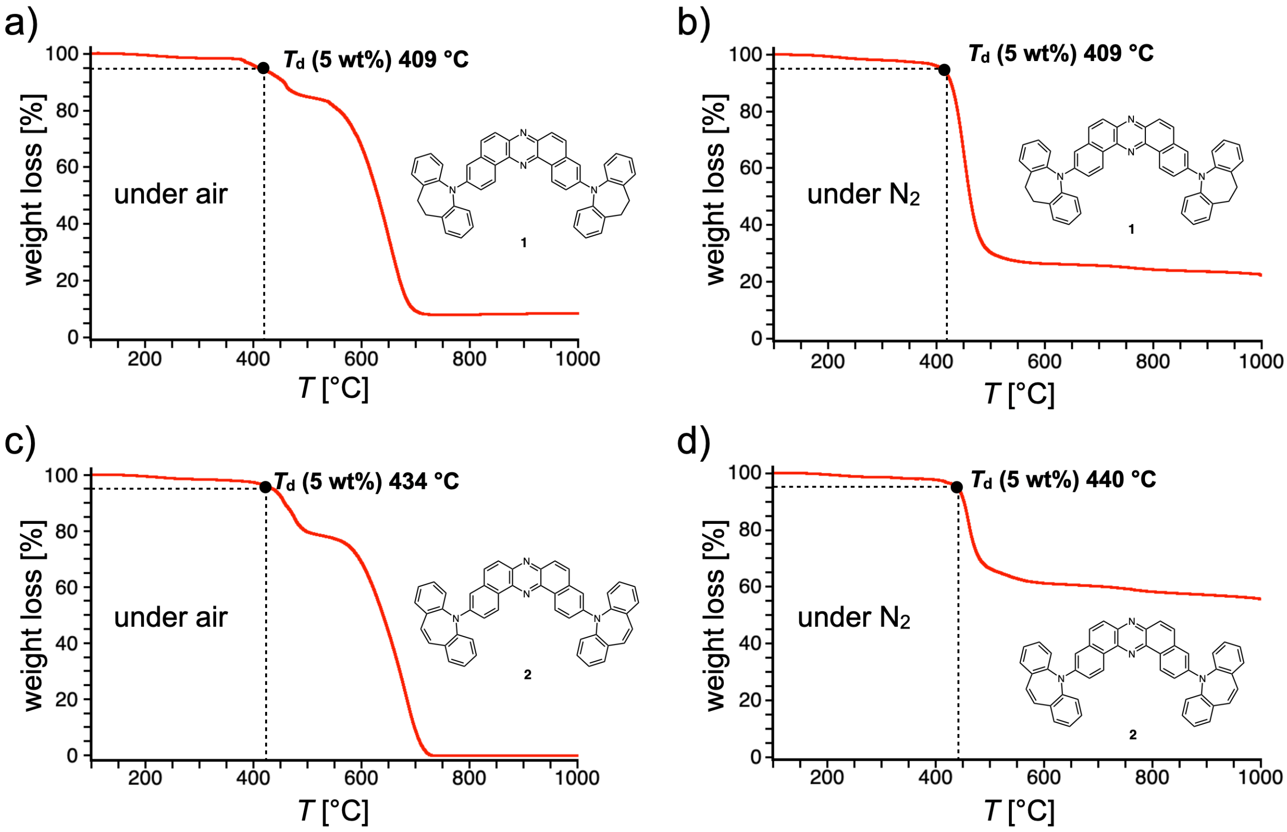

Figure S6. TGA profiles of (a) **1** under air, (b) **1** under N_2 gas, (c) **2** under air, and (d) **2** under N_2 gas.

Copies of ^{1}H and ^{13}C NMR

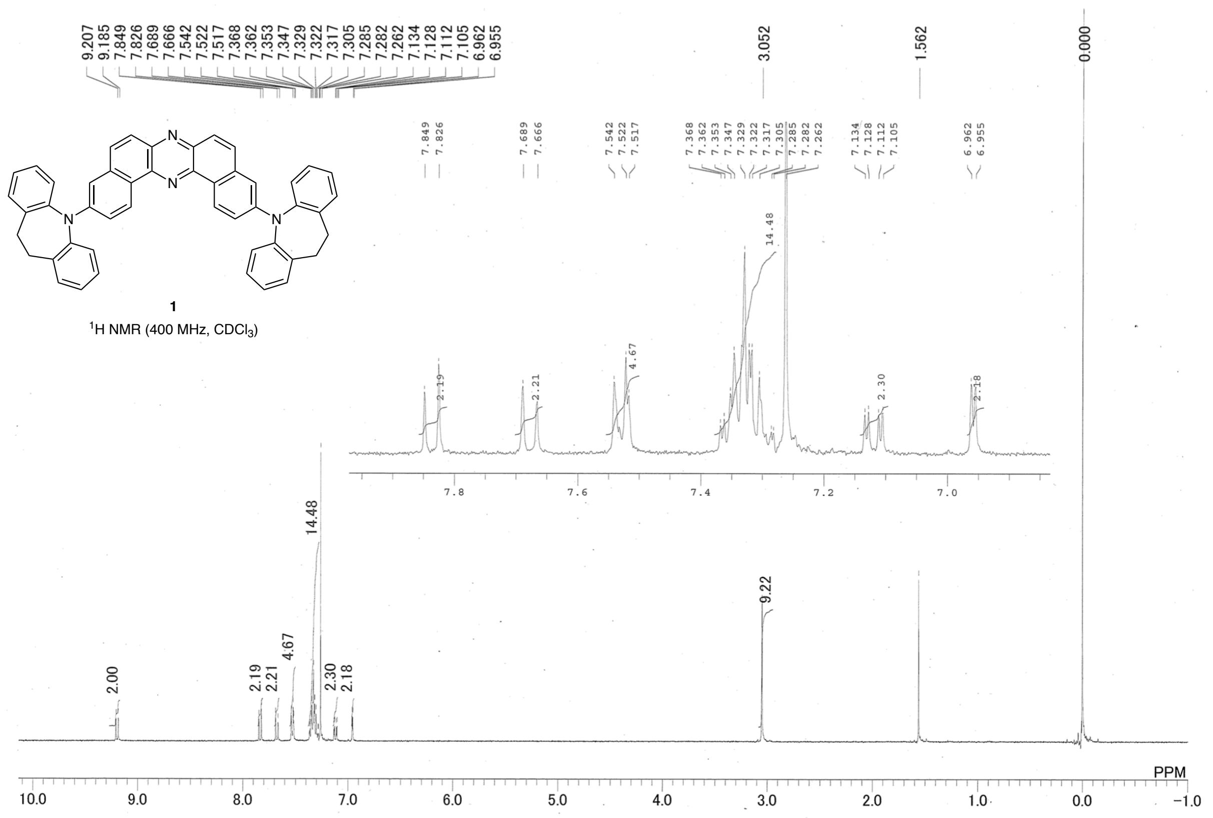

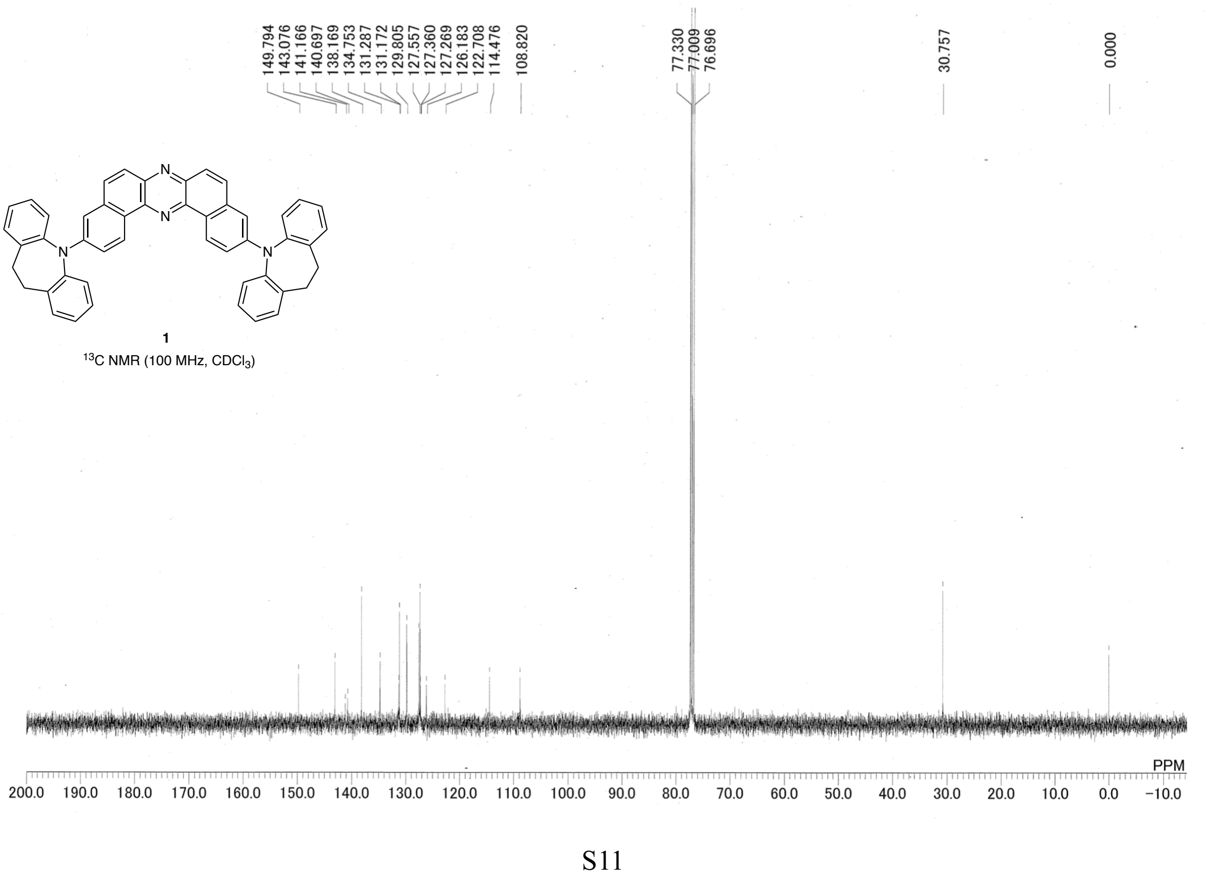

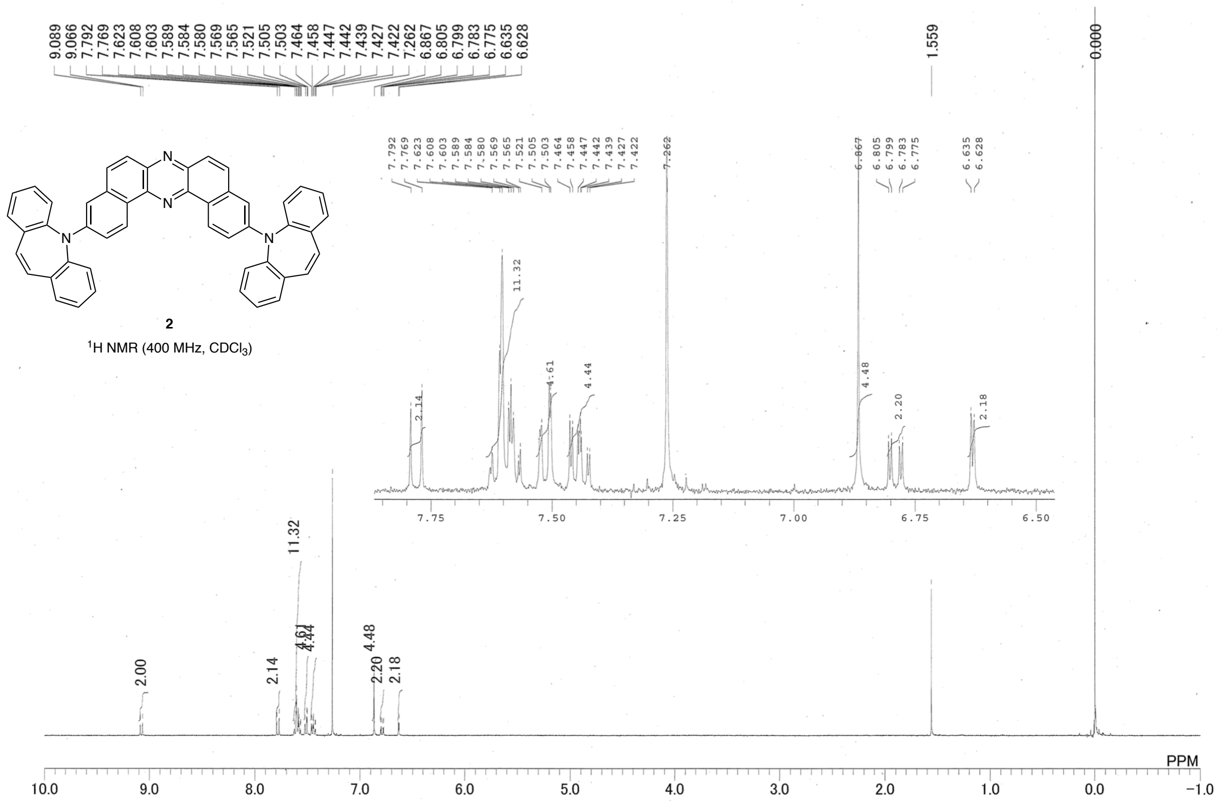

2

^{1}H NMR (400 MHz, $CDCl_3$)

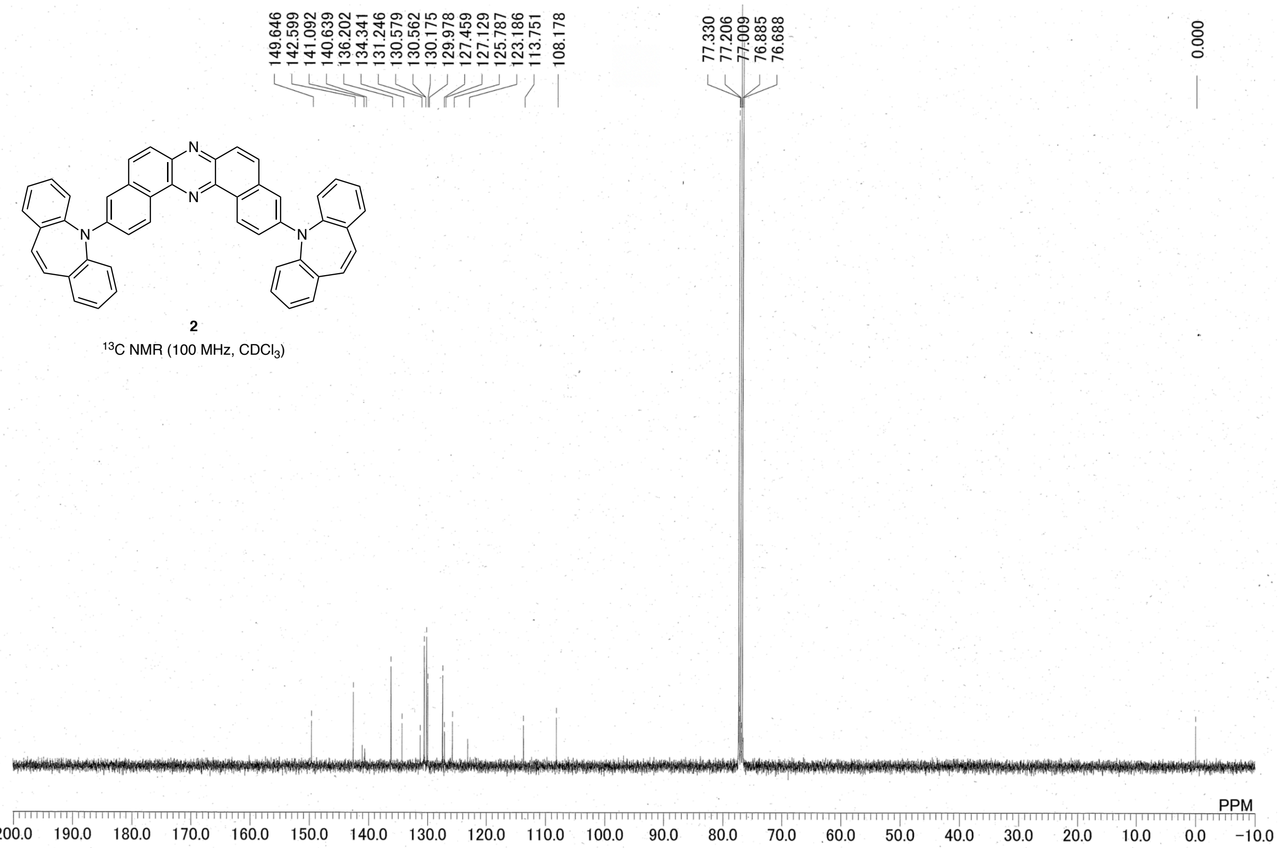

2

^{13}C NMR (100 MHz, $CDCl_3$)

Theoretical Calculations

Table S1. Energies of different conformations relative to the most stable conformer (ax-ax). Calculated at the PBE0/cc-pVDZ in a vacuum.

Conformation	Relative energy [eV]	
	**1**	**2**
**ax-ax**	0.00	0.00
**eq-ax**	0.22	0.32
**eq-eq**	0.49	0.67

Table S2. Energies of molecules in different oxidation states relative to the most stable conformer (ax-ax). Calculated at the PBE0/cc-pVDZ level using PCM solvation model for DCM.

	Relative energy [eV]					
	**1**			**2**		
	**ax-ax**	**eq-ax**	**eq-eq**	**ax-ax**	**eq-ax**	**eq-eq**
**neutral**	0.00	0.27	0.56	0.00	0.34	0.69
**cation**	0.00	0.13	0.46	0.00	0.43	0.76
**dication**	0.00	0.04	0.10	0.00	0.04	0.09
**anion**	0.00	0.09	0.23	0.00	0.18	0.38

Table S3. First (IP) and second (IP2) ionization potentials and electron affinity of the ax-ax conformers for compounds **1** and **2**.

	Energy [eV]	
	**1**	**2**
**IP**	5.32	5.36
**IP2**	5.70	5.76
**EA**	2.43	2.42

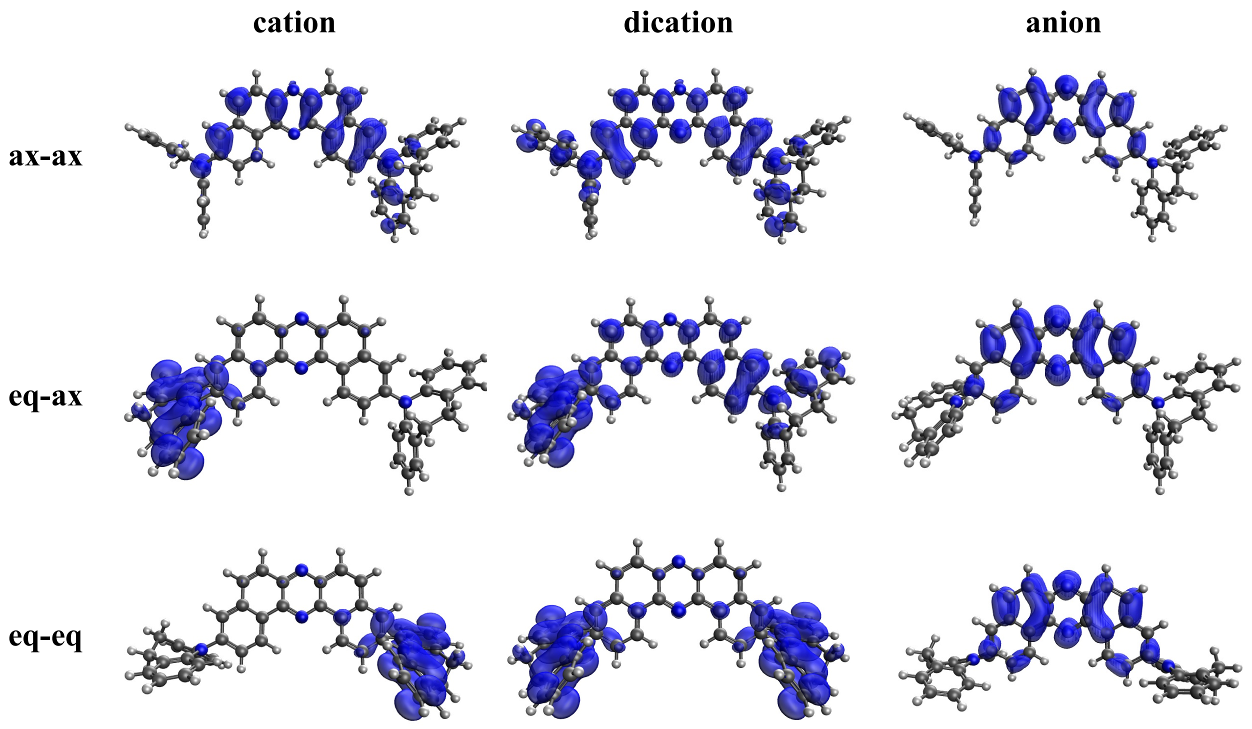

Figure S7. Spin densities of cation, diradical dication, and anion of **1** in different conformations.

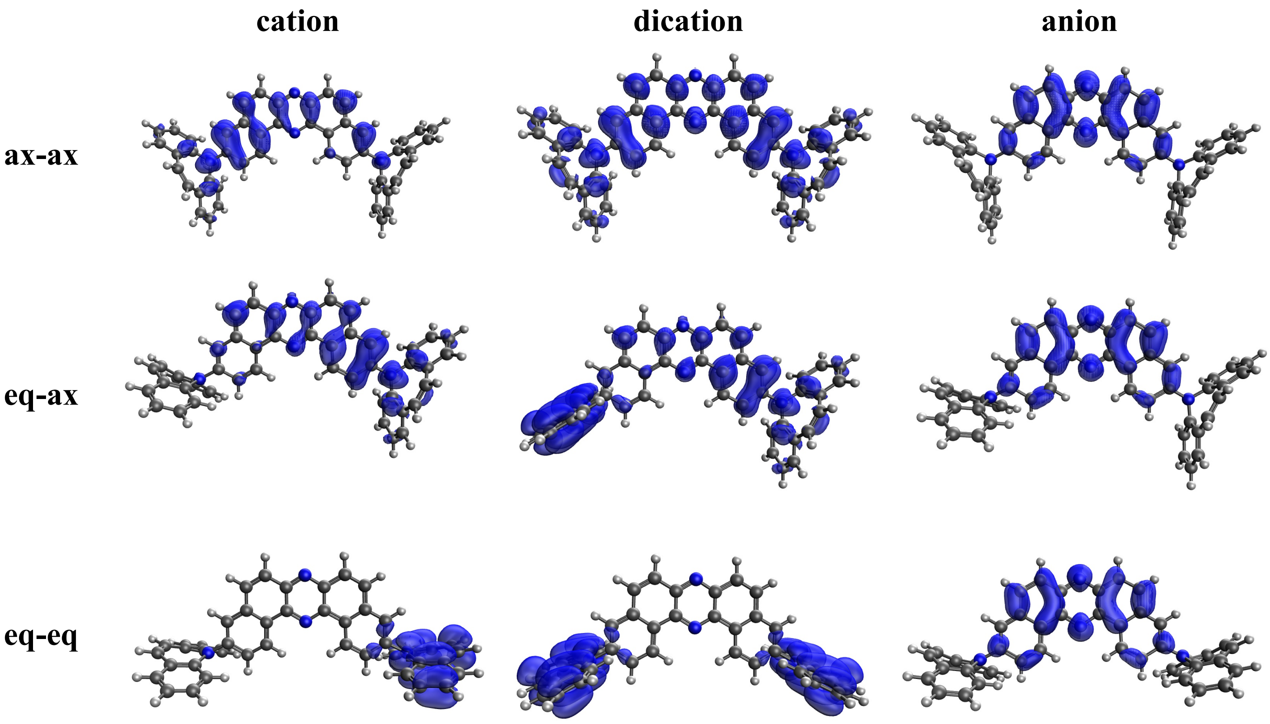

Figure S8. Spin densities of cation, diradical dication, and anion of **2** in different conformations.

Table S4. Excitation energies calculated at the ground state geometry at the ω*PBE/cc-pVDZ level, using state-specific PCM solvation model.

**Compound**	**Conformation**	**Excitation energy at the S_0 geometry [eV]**					
		S_1	S_2	S_3	T_1	T_2	T_3
**1**	**ax-ax**	2.94	3.16	3.18	2.38	2.47	2.71
	**eq-ax**	2.86	2.96	3.15	2.33	2.58	2.67
	**eq-eq**	2.70	2.81	3.15	2.53	2.59	2.64
**2**	**ax-ax**	2.97	3.03	3.08	2.41	2.49	2.70
	**eq-ax**	2.82	2.95	2.96	2.37	2.41	2.70
	**eq-eq**	2.82	2.85	3.10	2.42	2.43	2.58

Table S5. Energies of different electronic states at the ground state geometry relative to the most stable conformer (ax-ax).

	**Relative energy at the S_0 geometry [eV]**					
	**1**			**2**		
	**ax-ax**	**eq-ax**	**eq-eq**	**ax-ax**	**eq-ax**	**eq-eq**
**S_0**	0.00	0.29	0.60	0.00	0.25	0.52
**S_1**	0.00	0.21	0.36	0.00	0.10	0.37
**T_1**	0.00	0.23	0.74	0.00	0.21	0.54

Table S6. Excitation energies calculated at the S_1 excited state geometry at the ω*PBE/cc-pVDZ level, using state-specific PCM solvation model. *The starting eq-eq conformation of **2** relaxed to eq-ax during the excited-state optimization.

**Compound**	**Conformation**	**Excitation energy at the S_1 geometry [eV]**					
		S_1	S_2	S_3	T_1	T_2	T_3
**1**	**ax-ax**	2.69	2.92	3.13	2.21	2.40	2.58
	**eq-ax**	2.55	2.63	3.11	2.03	2.27	2.61
	**eq-eq**	2.18	2.95	3.07	2.21	2.40	2.58
**2**	**ax-ax**	2.05	2.76	2.81	2.04	2.23	2.37
	**eq-ax**	1.89	2.15	2.84	1.30	2.29	2.48
	**eq-eq***	1.87	2.13	2.83	1.28	2.28	2.48

Table S7. Energies of different electronic states at the S1 excited state geometry relative to the ax-ax conformer. *The starting eq-eq conformation of **2** relaxed to eq-ax during the excited-state optimization.

	**Relative energy at the S_1 geometry [eV]**					
	**1**			**2**		
	**ax-ax**	**eq-ax**	**eq-eq**	**ax-ax**	**eq-ax**	**eq-eq***
S_0	0.00	0.30	0.58	0.00	0.38	0.38
S_1	0.00	0.16	0.07	0.00	0.22	0.22
T_1	0.00	0.21	0.67	0.00	-0.37	-0.37

Table S8. Excitation energies calculated at the T_1 excited state geometry at the ω*PBE/cc-pVDZ level, using state-specific PCM solvation model. *The starting eq-eq conformation of **1** relaxed to ax-ax during the excited-state optimization.

**Compound**	**Conformation**	**Excitation energy at the T_1 geometry [eV]**					
		**S_1**	**S_2**	**S_3**	**T_1**	**T_2**	**T_3**
**1**	**ax-ax**	2.61	2.82	3.05	1.83	2.22	2.59
	**eq-ax**	2.54	3.06	3.10	1.82	2.43	2.58
	**eq-eq***	2.61	2.82	3.05	1.83	2.22	2.59
**2**	**ax-ax**	2.62	2.72	2.86	1.84	2.24	2.58
	**eq-ax**	1.77	2.05	2.42	1.11	2.28	2.48
	**eq-eq**	1.87	1.93	2.37	1.13	2.43	2.45

Table S9. Energies of different electronic states at the S_1 excited state geometry relative to the ax-ax conformer. *The starting eq-eq conformation of **1** relaxed to ax-ax during the excited-state optimization.

	**Relative energy at the S_1 geometry [eV]**					
	**1**			**2**		
	**ax-ax**	**eq-ax**	**eq-eq***	**ax-ax**	**eq-ax**	**eq-eq**
S_0	0.00	0.20	0.00	0.00	0.60	0.84
S_1	0.00	0.13	0.00	0.00	-0.28	-0.02
T_1	0.00	0.18	0.00	0.00	-0.13	0.13

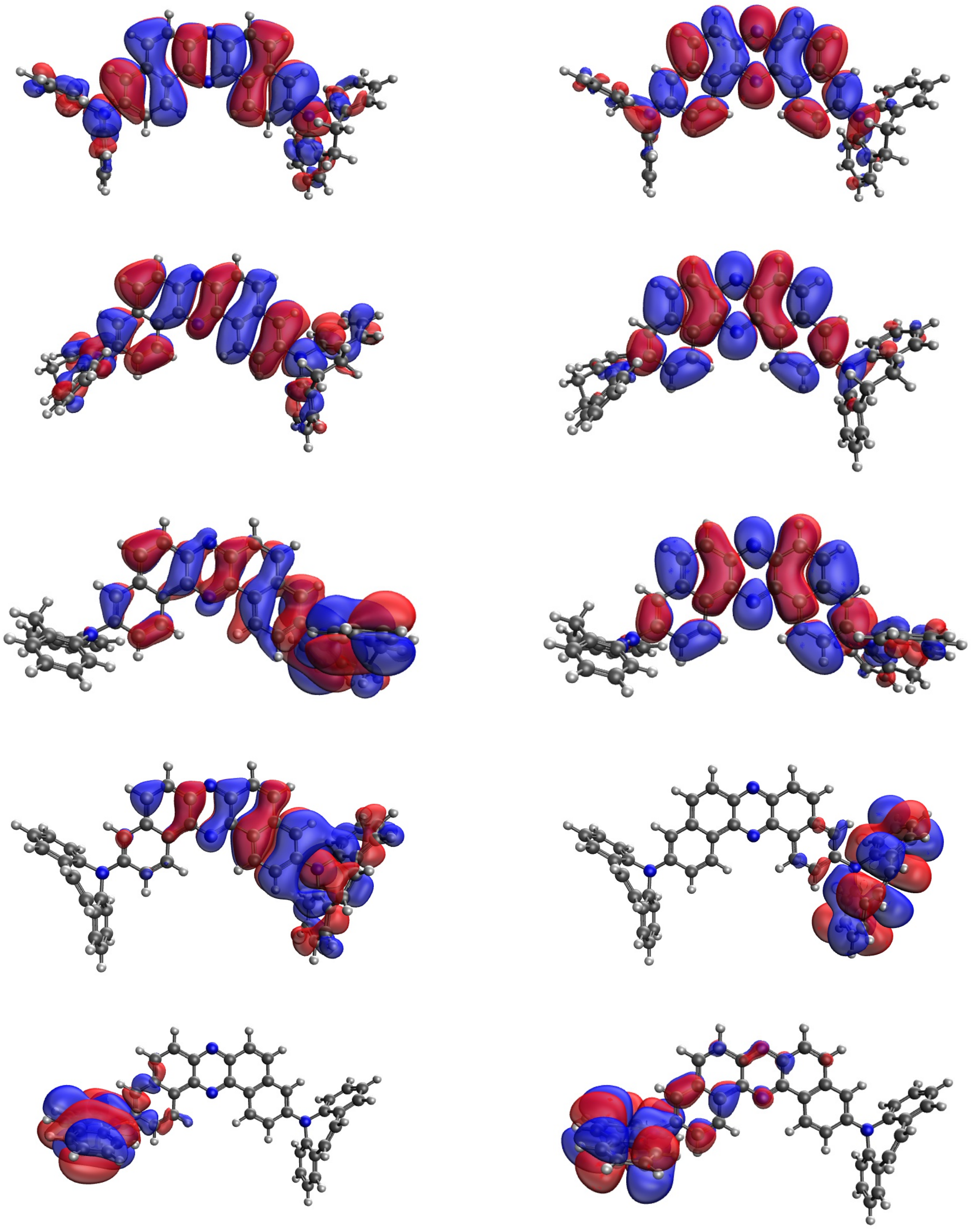

Figure S9. Natural transition orbitals for S_1 states calculated at the optimized S_1 geometry. *The starting eq-eq conformation of **2** relaxed to eq-ax during the excited-state optimization.

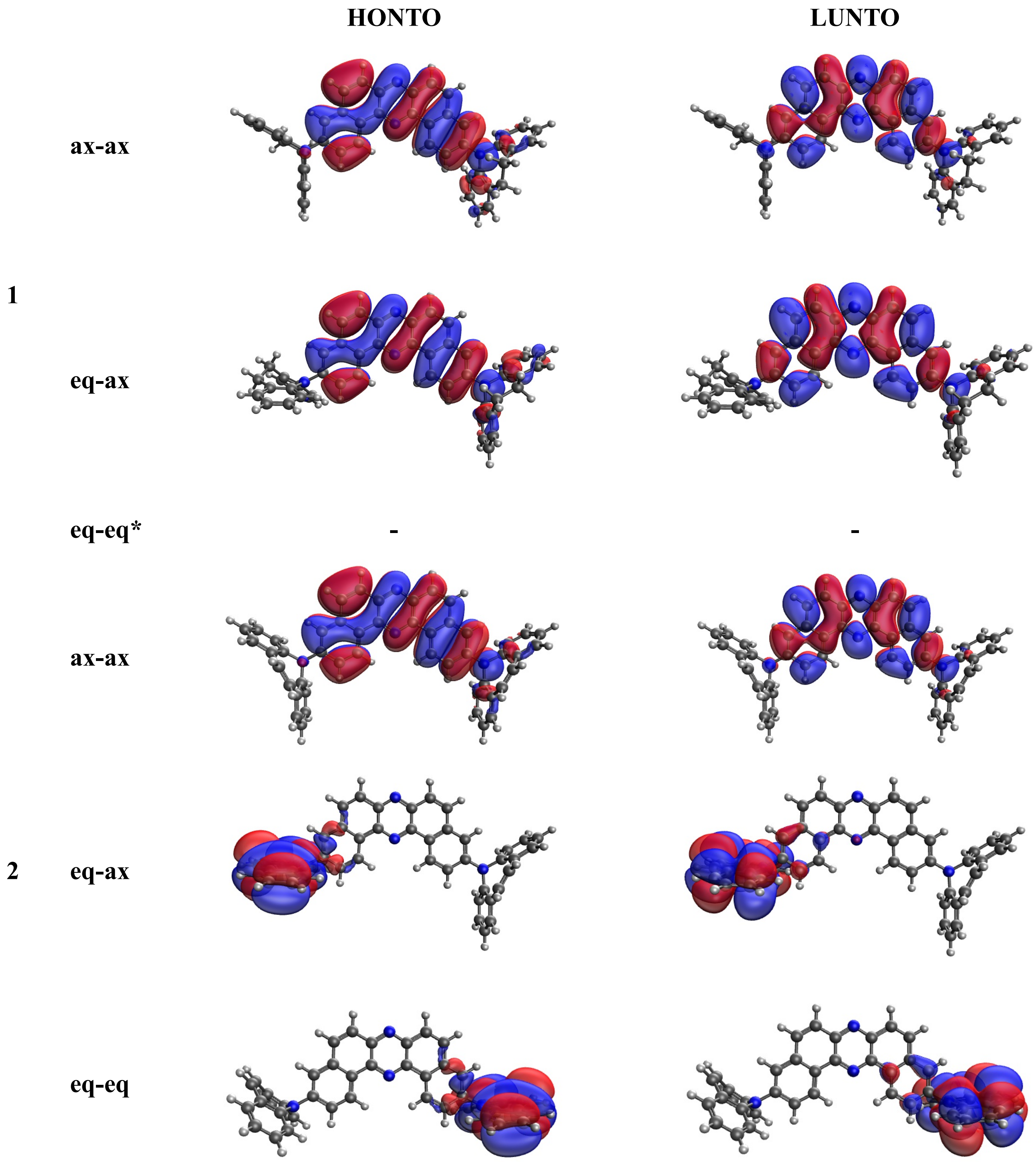

Figure S10. Natural transition orbitals for T_1 states calculated at the optimized T_1 geometry. *The starting eq-eq conformation of **1** relaxed to ax-ax during the excited-state optimization.

References

S1) P. Data, P. Pander, M. Okazaki, Y. Takeda, S. Minakata, A. P. Monkman, *Angew. Chem. Int. Ed.* **2016**, *55*, 5739–5744.

S2) G. te Velde, F. M. Bickelhaupt, E. J. Baerends, C. F. Guerra, S. J. A. van Gisbergen, J. G. Snijders, T. Ziegler, *J. Comput. Chem.* **2001**, *22*, 931–967.

S3) Y. Shao, Z. Gan, E. Epifanovsky, A. T. B. Gilbert, M. Wormit, J. Kussmann, A. W. Lange, A. Behn, J. Deng, X. Feng, D. Ghosh, M. Goldey P. R. Horn, L. D. Jacobson, I. Kaliman, R. Z. Khaliullin, T. Kús, A. Landau, J. Liu, E. I. Proynov, Y. M. Rhee, R. M. Richard, M. A. Rohrdanz, R. P. Steele, E. J. Sundstrom, H. L. Woodcock III, P. M. Zimmerman, D. Zuev, B. Albrecht, E. Alguire, B. Austin, G. J. O. Beran, Y. A. Bernard, E. Berquist, K. Brandhorst, K. B. Bravaya, S. T. Brown, D. Casanova, C.-M. Chang, Y. Chen, S. H. Chien, K. D. Closser, D. L. Crittenden, M. Diedenhofen, R. A. DiStasio Jr., H. Dop, A. D. Dutoi, R. G. Edgar, S. Fatehi, L. Fusti-Molnar, A. Ghysels, A. Golubeva-Zadorozhnaya, J. Gomes, M. W. D. Hanson-Heine, P. H. P. Harbach, A. W. Hauser, E. G. Hohenstein, Z. C. Holden, T.-C. Jagau, H. Ji, B. Kaduk, K. Khistyaev, J. Kim, J. Kim, R. A. King, P. Klunzinger, D. Kosenkov, T. Kowalczyk, C. M. Krauter, K. U. Lao, A. Laurent, K. V. Lawler, S. V. Levchenko, C. Y. Lin, F. Liu, E. Livshits, R. C. Lochan, A. Luenser, P. Manohar, S. F. Manzer, S.-P. Mao, N. Mardirossian, A. V. Marenich, S. A. Maurer, N. J. Mayhall, C. M. Oana, R. Olivares-Amaya, D. P. O'Neill, J. A. Parkhill, T. M. Perrine, R. Peverati, P. A. Pieniazek, A. Prociuk, D. R. Rehn, E. Rosta, N. J. Russ, N. Sergueev, S. M. Sharada, S. Sharmaa, D. W. Small, A. Sodt, T. Stein, D. Stück, Y.-C. Su, A. J. W. Thom, T. Tsuchimochi, L. Vogt, O. Vydrov, T. Wang, M. A. Watson, J. Wenzel, A. White, C. F. Williams, V. Vanovschi, S. Yeganeh, S. R. Yost, Z.-Q. You, I. Y. Zhang, X. Zhang, Y. Zhou, B. R. Brooks, G. K. L. Chan, D. M. Chipman, C. J. Cramer, W. A. Goddard III, M. S. Gordon, W. J. Hehre, A. Klamt, H. F. Schaefer III, M. W. Schmidt, C. D. Sherrill, D. G. Truhlar, A. Warshel, X. Xua, A. Aspuru-Guzik, R. Baer, A. T. Bell, N. A. Besley, J.-D. Chai, A. Dreuw, B. D. Dunietz, T. R. Furlani, S. R. Gwaltney, C.-P. Hsu, Y. Jung, J. Kong, D. S. Lambrecht, W. Liang, C. Ochsenfeld, V. A. Rassolov, L. V. Slipchenko, J. E. Subotnik, T. Van Voorhis, J. M. Herbert, A. I. Krylov, P. M. W. Gill, and M. Head-Gordon. *Advances in molecular quantum chemistry contained in the Q- Chem 4 program package.*
